# Evaluating the costs of cholera illness and cost-effectiveness of a single dose oral vaccination campaign in Lusaka, Zambia

**DOI:** 10.1371/journal.pone.0215972

**Published:** 2019-05-31

**Authors:** Tannia Tembo, Michelo Simuyandi, Kanema Chiyenu, Anjali Sharma, Obvious N. Chilyabanyama, Clara Mbwili-Muleya, Mazyanga Lucy Mazaba, Roma Chilengi

**Affiliations:** 1 Centre for Infectious Disease Research in Zambia, Lusaka, Zambia; 2 Lusaka District Health Office/ Ministry of Health, Lusaka, Zambia; 3 Zambia National Public Health Institute, Lusaka, Zambia; Ministry of Health and Sports, MYANMAR

## Abstract

**Introduction:**

In 2016, for the very first time, the Ministry of Health in Zambia implemented a reactive outbreak response to control the spread of cholera and vaccinated at-risk populations with a single dose of Shancol—an oral cholera vaccine (OCV). This study aimed to assess the costs of cholera illness and determine the cost-effectiveness of the 2016 vaccination campaign.

**Methodology:**

From April to June 2017, we conducted a retrospective cost and cost-effectiveness analysis in three peri-urban areas of Lusaka. To estimate costs of illness from a household perspective, a systematic random sample of 189 in-patients confirmed with *V*. *cholera* were identified from Cholera Treatment Centre registers and interviewed for out-of-pocket costs. Vaccine delivery and health systems costs were extracted from financial records at the District Health Office and health facilities. The cost of cholera treatment was derived by multiplying the subsidized cost of drugs by the quantity administered to patients during hospitalisation. The cost-effectiveness analysis measured incremental cost-effectiveness ratio—cost per case averted, cost per life saved and cost per DALY averted—for a single dose OCV.

**Results:**

The mean cost per administered vaccine was US$1.72. Treatment costs per hospitalized episode were US$14.49–US$18.03 for patients ≤15 years old and US$17.66–US$35.16 for older patients. Whereas households incurred costs on non-medical items such as communication, beverages, food and transport during illness, a large proportion of medical costs were borne by the health system. Assuming vaccine effectiveness of 88.9% and 63%, a life expectancy of 62 years and Gross Domestic Product (GDP) per capita of US$1,500, the costs per case averted were estimated US$369–US$532. Costs per life year saved ranged from US$18,515–US$27,976. The total cost per DALY averted was estimated between US$698–US$1,006 for patients ≤15 years old and US$666–US$1,000 for older patients.

**Conclusion:**

Our study determined that reactive vaccination campaign with a single dose of Shancol for cholera control in densely populated areas of Lusaka was cost-effective.

## Introduction

Cholera is a significant global health problem and is endemic in Africa and Asia where access to improved water and sanitation is inadequate [1]. It can be acquired through ingestion of food or water contaminated with bacterium *Vibrio Cholerae* serogroup O1 or O139. Cholera disease is characterized by a sudden onset of acute watery diarrhea that can rapidly lead to death by severe dehydration, if untreated [2,3].

An estimated 1.4 billion people are at risk of cholera worldwide. Of the 132,121 cases reported in 2016, 54% were from Africa, 32% from Hispaniola and 13% from Asia [1,4]. Zambia first reported cholera outbreaks in 1978 and has suffered large cholera epidemics with around 13,000 cases reported in 1991 and over 11,000 in 1992 and 1999 [5]. From 1999 to 2013, major outbreaks have been reported every year in the country’s capital, Lusaka [6]. The 2016 outbreak on which this paper is based recorded a total of 1,079 cases and 20 deaths. Lusaka experienced another cholera outbreak in 2017–18 with an estimated 5,414 cumulative cases and a Case Fatality Ratio (CFR) of 1.8% [7–9].

Prolonged and frequent outbreaks can be devastating and often dramatically impact economies thereby causing increased expenditure on treatment, declines in income for cholera patients and their households, reduced exports and loss of tourism revenue [10]. In 2005, the 125,018 cases of cholera notified to the World Health Organization (WHO) resulted in a real total economic loss of US$38.9–US$64.2 million [11]. In 1997, the average household and health systems costs of illness per episode were estimated at US$104 as compared to US$43 estimated by hospital-based studies in 2005 [2]. Cost-effective measures to prevent and control cholera should be implemented to mitigate such economic losses.

A multifaceted approach is key to preventing and controlling cholera outbreaks, including improved water, sanitation and hygiene (WASH), health education and promotion, social mobilization, surveillance and timely treatment with Oral Rehydration Salts (ORS), intravenous (IV) fluids, antibiotics and, Zinc supplements for children aged 6 months to 5 years [3,12,13]. However, since the improvement of WASH remains difficult to achieve in many endemic countries, Oral Cholera Vaccines (OCVs) have been recommended as a supplementary, cost-effective and more immediate option for cholera control and prevention [2,3]. Previous results have demonstrated that OCVs confer significant herd protection over several years [14].

In 2001, the WHO prequalified Dukoral (SBLVaccine, Sweden) for purchase by United Nations (UN) agencies [15]. Since then, several studies have documented the protective effect of OCVs [16–18] and the feasibility of mass vaccination campaigns [19,20]. Shancol (Shantha Biotechnics Limited, Hyderabad, India), a killed whole-cell OCV pre-qualified in 2011, is said to be easier to deliver in challenging field conditions and less costly because it does not contain the cholera toxin and does not require co-administration with an oral buffer [15,21].

Vaccine purchase and delivery cost per-double dose of Shancol was estimated at US$0.49–US$0.97 between 2008 and 2016. Similarly, the total cost per fully vaccinated individual was estimated at US$1.13 and increased to US$8.75 in resource-poor settings. In Malawi, the cost of vaccine purchase and shipment per fully vaccinated person was US$5.20 in 2016 and the financial cost for delivering the vaccine was US$1.94. Personnel costs accounted for almost 71% of the total economic costs of vaccine delivery [22].

Assuming 50% vaccine coverage among individuals aged ≥2years, a reactive vaccination campaign in Zimbabwe could have averted 1,320 deaths and 23,650 Disability Life Adjusted Years (DALYs) in 2008–2009 [23]. Cholera vaccinations among 1–14 year olds every three years would be the most cost-effective, reducing incidence by 45% and costing US$823 for single dose vials per DALY averted as compared to more than 90% reduction in incidence for older persons, but less cost-effective at US$894–US$1,234 per DALY [24].

Even though cholera outbreaks occur periodically in Zambia, there is a dearth of information on the costs of cholera illness and cost-effectiveness of OCV campaigns. The purpose of this study was to assess the cost of cholera illness from a household and health system perspective and determine if the single dose OCV campaign that was implemented for the first time in response to the 2016 cholera outbreak in Lusaka was cost-effective. As the country structures ongoing efforts towards developing comprehensive and integrated plans for controlling and preventing future cholera outbreaks, the results of this analysis could help the Ministry of Health (MoH) to decide whether or not to use OCVs as a reactive measure.

## Materials and methods

### Study design and setting

This retrospective cost and cost-effectiveness study was conducted in Lusaka’s Bauleni, Chawama and Kanyama compounds, densely populated peri-urban areas with high cholera incidence. Between April and June 2017, we conducted interviews with participants identified from health facility registers. Costs were collected retrospectively from financial records at health facilities and the Lusaka District Health Office (LDHO).

The City of Lusaka is situated in the central part of Zambia on the Central African plateau and lies 1,280 meters above sea level with a surface area of 360 square kilometers. It has a population density of 5,328 persons per square kilometer and an estimated total population of 1.9 million [25]. Health care needs of residents are catered for by public and private health facilities. In the event of a cholera outbreak, specific public health facilities become designated Cholera Treatment Centres (CTCs)–specialized isolation wards for cholera patients.

The 2016 cholera outbreak affected populations in nine peri-urban locations in Lusaka district after a four-year absence, prompting increased concern over vulnerability to infection of approximately 600,000 people living in unsanitary conditions. To prevent further transmission of cholera and shorten the duration of the epidemic, the MoH with support from Médecins Sans Frontières (MSF) and the WHO, mounted a reactive outbreak response using community-based mass vaccination campaigns and vaccinated 424,100 (73.4%) persons aged one year and older with a single dose of Shancol [26]. The OCV was used as a supplement efforts to improve water availability and quality.

### Data collection procedures

A research team consisting of an investigator and six Research Assistants (RAs) collected costs incurred by households and the health system. The RAs were trained for two days and provided with Standard Operating Procedures (SOPs) containing step-by-step instructions on how to administer the questionnaires to patients or their caregivers and health care providers and extract data from identified sources. Table 1 summarises expenditure variables and sources of health system, household and vaccine delivery and campaign costs.

**Table 1:** Expenditure components and sources of data for health system and household costs.

Cost component	Description of Costs	Source
**Vaccination delivery and campaign costs**		
Vaccine delivery	Vaccine purchase, freight, insurance and cold-chain management, transport, storage	Reports from LDHO and MSF
Social mobilisation and sensitisation	Training (conference package, materials), public address, dissemination and leaflets	
Staff incentives	Transport, lunch and per diems	Finance and Human Resources records
Other	Motor vehicles, waste management, stationery, communication, equipment and consumables	Reports from LDHO and MSF
**Health system costs**		
Fixed costs	Ward supplies—Tents, beds, blankets, buckets, waste bins, sharp boxes	Reports from LDHO and MSF
	Allowances	Financial records,
		interviews with staff at CTCs
Variable costs	Personal protective equipment—Work-suits, gumboots, gloves, aprons, laboratory coats	Stock cards
	Water sampling kits	
	Laboratory supplies—RDT Bioline tests, reagents, swabs, cannulas, needles	
	Pharmacy supplies—Antibiotics, ORS, fluids	Bin cards and invoices from MSL
**Patient costs**		
Direct	Medical (consultation, drugs, consumables) and non-medical (accommodation, communication, transport) costs	Interviews with confirmed cases
Indirect	Productivity losses and opportunity costs	

To enable mobile collection of data, forms were created in .xml using Open Data Kit (ODK), an open-source suite of tools, and loaded onto tablets before interviews were held with patients or caregivers and health care workers. For quality improvement purposes, both the household and health facility questionnaires were piloted with selected patients or caregivers and health care workers. Prior to the interviews, written informed consent was obtained from health care providers, patients or caregivers (for patients aged below 18 years). At the end of each interview, forms were reviewed for accuracy and completeness before being committed to a central server. Since data were collected more than four months after the identified patients had been treated for cholera and discharged from a CTC, out-of-pocket costs provided by interviewees were verified with reliable sources such as drug catalogues, public transportation fare charts and vendors’ price lists.

#### Household costs of cholera

To determine the sample size for estimating out-of-pocket costs, a standard deviation of US$40 was estimated as the mean household cost for treating cholera. The following formula was applied:

$$n=4{\left(standard\;deviation\right)}^{2}/a^{2}\;with\;a=6\;[2].$$

A systematic random sample of 189 (60, 67 and 62 from Bauleni, Chawama and Kanyama health centres, respectively) patients were selected from the 1,079 patients confirmed to have *V*. *cholera* 01 or 0139 by culture of a stool specimen. Patient information was extracted for every 5^th^ patient admitted to a CTC and documented in a register.

Community Health Workers (CHWs—members of the community who often voluntarily support trained health workers and provide basic health care to communities) contacted the identified patients and scheduled appointments for one-on-one interviews at the patient’s home or a place of the patient’s convenience. On a subsequent day, a research team met the patients as scheduled and obtained direct and indirect costs using the closed-ended household questionnaire. If the identified patient was below 18 years of age, a caregiver was interviewed.

#### OCV vaccine delivery and campaign costs

Due to unavailability of sufficient doses in the global emergency stockpile to cover the entire at-risk population, a single-dose of Shancol was administered to people living in the most affected peri-urban areas [27]. Vaccination delivery and campaign costs were therefore collected for a single dose OCV. Costs were obtained from the LDHO and MSF since they had provided financial and technical support for the mass vaccination campaign. The costs derived by Poncin et al., 2018 have been used to provide estimates of the costs of vaccine delivery and mass vaccination campaign as incurred by MSF [26].

#### Health systems costs of cholera

To estimate costs from a health system perspective, a research team interviewed nurse-midwives, medical doctors or Environmental Health Technicians (EHTs—staff working in health facilities to identify and rectify environmental health issues) working at CTCs and the LDHO. Staff at the LDHO were interviewed about central level costs. Staff at CTCs provided information on procedures for setting up CTCs and managing hospitalised cholera patients. Using an ingredients-based approach, direct and indirect costs were extracted from financial records and cash registers at health facilities and the LDHO [28]. Patient records were reviewed for length of hospital stay and drugs administered to patients during hospitalisation and after discharge. Costs of drugs were obtained from the Medical Stores Limited (MSL)—an autonomous government agency that provides drugs to public health facilities at a subsidised price [29].

#### Cost-effectiveness analyses

This study used the Vaccine Introduction Cost-effectiveness (VICE) calculator to estimate the cost-effectiveness of the 2016 OCV campaign with a single dose of Shancol. The VICE calculator is a Microsoft Excel-based tool that was specifically developed to simplify the estimation of cost-effectiveness of OCVs in various settings by using variables that influence the cost-effectiveness of the vaccine. It is capable of highlighting important nuances when different values for individual variables are entered and automatically calculates cost-per-DALY averted, cost per-case- averted and cost-per-death averted [30]. To calculate the resulting ICERs, standardized parameters such as disability weights, discount rate, case fatality ratio (CFR), GDP and vaccine protection in Zambia [31] and Haiti [3,32] were obtained from literature [18]. Other parameters including length of hospital stay, duration of illness, incidence, CFR and treatment costs were derived from this analysis. In place of age weights, a single, homogeneous population was used to compute the cost-effectiveness measures.

In Zambia, as is the case in other countries, drugs are administered to hospitalized cholera patients according to age and severity of infection. Treatment costs were therefore computed for two categories of patients—pediatric patients (≤years old) and older patients (≥15 years) [1,33]–and used as base-estimates for calculating ICERs. The analysis used Zambia’s specific life expectancy (LE) as derived by the World Bank Group [34].

The primary outcome measures of the cost-effectiveness analysis (CEA) were ICERs per DALY averted, per case and death averted. The net vaccination cost was used as the numerator of the ICER and was calculated by subtracting the costs of illness averted from the total vaccination costs at a discount rate of 3% [17]. To be considered cost-effective, derived costs per DALYs averted should have been less than three times the country’s GDP per capita valued of $1,500 in 2016 [28,30,33,34,35].

Table 2 shows parameter values assumed for computing the cost-effectiveness of a vaccination campaign using a single dose of Shancol.

**Table 2:** Parameter values assumed for computing cost-effectiveness of a vaccination campaign using a single dose of Shancol in Zambia.

Description of Input	Value	Source of Data
Life expectancy at infection	62 years	The World Bank, 2018
Annual discount rate	3%	Troeger et al., 2014 [30]
Duration of immunity	2 years	WHO, 2017 [3]
Disability weight	0.2%	Salomon et al., 2012 [36]
GDP per Capita	US$1,500	The World Bank [34]
Vaccine effectiveness	89%	Ferreras et al., 2018 [31]
	63%	WHO, 2017; Ivers et al., 2016 [3,32]
Duration of cholera illness	3 days	Derived from this analysis
Incidence	2.5%	Derived from this analysis
Case fatality rate	1.9%	Derived from this analysis
Vaccine purchase cost/dose	US$1.31	Derived from this analysis
Vaccine delivery/ dose	US$0.41	Poncin et al., 2018 [26]
OCV coverage	73.4%	Poncin et al., 2018 [26]
**Direct treatment costs by age group**		
Patients ≤15 years (Lower limit)	US$14.49	Derived from this analysis
Patients ≤15 years (Upper limit)	US$18.03	Derived from this analysis
Patients ≥15 years (Lower limit)	US$17.66	Derived from this analysis
Patients ≥15 years (Upper limit)	US$35.16	Derived from this analysis

To determine the effects of changes in direct cost of treatment, days of illness, GDP, vaccine efficacy, duration of protection and vaccine purchase price on cost-effectiveness, sensitivity analysis was performed using Microsoft Excel (MS Excel, 2016).

### Data management and analysis

This study used a cost of illness approach forwarded by Rice [37] and adopted the six suggested cost components for analysing costs of cholera in the WHO African Region which include: (1) hotel, hospital and health centre costs; administration, health personnel remunerations, in-service training, per diem and transport, materials, utilities (electricity, water, telephone and postage), maintenance (of vehicles, equipment and buildings), and capital costs; (2) diagnosis, (3) medicines, (4) costs borne by households, (5) productivity losses, and (6) other losses [10].

The Choltool, a standardised Microsoft Excel-based cholera cost calculator developed by International Vaccine Institute (IVI) [38,39], was adapted for estimating vaccine delivery, mass vaccination campaign and health systems costs. Costs incurred by households and the health system were collected in local currency units (LCUs) and converted to United States Dollars (US$) using the exchange rate at midpoint of 2016. The values were adjusted for inflation. Household and health system data were analysed using Stata 14.2 (StataCorp, College Station, Texas, USA).

### Ethics statement

This study protocol was approved by the University of Zambia Biomedical Research Ethics Committee (UNZA BREC-Reference Number 017-10-16) and the Zambia National Health Research Authority (ZNHRA). Permission to visit the health facilities was obtained from MoH and LDHO. Study participants were not given any incentives for participation. Individual written informed consent was obtained from all participants. All data were handled with strict confidentiality and de-identified by assigning individual unique identifiers before data was analysed.

## Results

### Household costs of illness

Table 3 represents selected characteristics of study participants and caregivers. Approximately 16.4% of the sampled patients were children aged between 2 and 15 years old. Of the 189 patients interviewed, 84 (44.4%) did not have a monthly income as they were either in school (10.6%), involved in leisure or play time (13.2%) or performing housework (20.6%). Of the 105 (55.6%) respondents who reported earning an income (results not shown), 45 (42.8%) earned US$0.10–US$48.49 (<US$1.50 a day) per month while 60 (57.1%) earned more. At least 89 (47.1%) of the households had self-employed persons. During cholera disease, approximately 11 (5.8%) households had lost between 1–3 days of income. Overall, 66.7% first sought care and treatment from sources other than CTCs or public health facilities.

**Table 3:** Selected characteristics of study participants and caregivers.

	Total (n = 189)	Percent (%)
**Sex**		
Male	66	34.9
Female	123	65.1
**Age**		
1–15 years	31	16.4
16–24 years	41	21.7
>24 years	117	61.9
**Education**		
Primary	53	28.0
Secondary	107	56.6
Tertiary	10	5.3
Never been to school	19	10.1
Employment*^a^		
Employed	16	8.5
Work for self	89	47.1
Housework	39	20.6
Other*^b^	45	23.8
**Source of drinking water**		
Private water source	18	9.5
Public water source	171	90.5
**Type of Toilet**		
Individual flushing toilet	6	3.2
Common flushing toilet	17	9.0
Pit latrines	166	87.8
**1st Place of visit before diagnosis**		
Cholera Treatment Centre	47	24.8
Public health centre*^c^	16	8.5
Other*^d^	126	66.7
**Patientcs health status**		
Recovered	182	96.3
Still sick with cholera	1	0.5
CFR*^e^	6	5.6

*^a^ Data does not show income brackets of respondents

*^b^ Includes patients in school or involved in leisure or play time

*^c^ Public health centre /post did not have an established CTC

*^d^ Includes visits to pharmacies, private health centres, traditional healers and use of low cost alcohol

*^e^ CFR calculated for the study sample size

### Household costs during hospitalisation

During hospitalisation, a few households reported paying for direct medical costs such as consultation (1.1%), drugs (0.5%) and laboratory tests (0.5%). The remaining households incurred non-medical costs, including communication (12.2%), food and beverages (14.8%) and transport (56.1%). Only a small proportion of respondents spent between US$9.8 and US$48.41 on transport (4.8%) and cell phone calls (2.1%). None of the respondents spent above US$96.81. Table 4 represents costs incurred during hospitalization.

**Table 4:** Costs incurred during hospitalisation.

	$0*^a^^,^ ^b^ (n = %)*c	$0.1–$9.7*^b^ (n = %)*c	$9.8–$48.41*^b^ (n = %)*c	$48.52–$96.81*^b^ (n = %)*c
Communication	166 (87.8)	19 (10.1)	4 (2.1)	0 (0.0)
Consultation	187 (98.9)	2 (1.1)	0 (0.0)	0 (0.0)
Drugs and supplies	188 (99.5)	1 (0.5)	0 (0.0)	0 (0.0)
Food and beverages	162 (85.7)	28 (14.8)	0 (0.0)	0 (0.0)
Laboratory tests	187 (98.9)	2 (1.1)	0 (0.0)	0 (0.0)
Transport	83 (43.9)	96 (50.8)	9 (4.8)	1 (0.5)

*^a^ Respondents did not spend any amount on cost categories during hospitalisation

*^b^ Local Currency Unit (LCU) converted to United States Dollar (US$) at an exchange rate of ZMW1:US$10.33 using mid-rate in 2016.

*^c^ N = Total number of patients (out of 189 patients interviewed) that incurred costs during hospitalisation

### Productivity losses

The majority of patients 134 (70.9%) stayed in hospital for 1 to 3 days while only 8 (4.2%) were admitted for more than seven days. At least 85 (45.0%) of the patients reported another 1–3 days of inactivity after being discharged from a CTC while only 13 (6.9%) said they were inactive for more than seven days. A total of 29 (15.3%) performed their normal activities immediately after discharge. Even though 123 (65.1%) households engaged helpers during the course of a member’s illness, only 2 (1.1%) paid US$0.1–US$9.7 for help rendered. To cope with lost income during illness, at least 53 (28%) of the households used their savings, 2.6% sold livestock and household assets, 31 (16.4%) borrowed money and 26 (13.8%) reduced their expenses (Table 5).

**Table 5:** Productivity losses during cholera outbreak.

	Total (n = 189)	Percent (%)
**Days of hospitalisation**		
1–3 days	134	70.9
4–7 days	47	24.9
>7 days	8	4.2
**Days of inactivity**		
0 days	29	15.3
1–3 days	85	45.0
4–7 days	62	32.8
>7 days	13	6.9
**Lost Income**		
1–3 days	11	5.8
4–7 days	22	11.6
>7 days	5	2.6
No lost income	151	79.9
**Help during Sickness**		
Yes	123	65.1
No	66	33.3
**Paid Helper**		
Yes	2	1.1
No	187	98.9
**Coping Strategy for Lost Income**		
Sold personal property	5	2.6
Borrowed money	31	16.4
Used savings	53	28.0
Reduced expenses	26	13.8
No coping strategy	74	39.2

### Vaccine delivery and OCV campaign costs

Vaccine delivery and campaign costs consisted of consumables, Information, Education and Communication (IEC) materials, vaccine purchase, insurance, shipment, storage, social mobilisation, staff allowances and trainings. The largest proportion of costs were reported for purchase of vaccines (80.2%) and staff incentives (6.7%). Other costs were reported as almost equally distributed among other items, including shipment (2.0%), vaccination consumables (2.6%), social mobilisation (1.6%), and transport (3.2%) [26].

### Health systems costs

The analysis in Table 6 shows that the largest proportion of costs incurred by health system was for the purchase of disinfectants (20.7%), followed by laboratory supplies (16.4%) and then drugs and ward supplies (16.1%). Other costs were distributed between personal protective clothing (14%), water sampling kits (5.7%) and miscellaneous items (8.5%).

**Table 6:** Total direct costs incurred by the Ministry of Health and health facilities during cholera outbreak.

Characteristic	Cost (US$)	Total (%)
Disinfectants	143 010	20.7
Personal protective equipment	96 214	14.0
Water sampling kits	38 265	5.7
Laboratory supplies	113 061	16.4
Rehydration fluids	16 881	2.5
Drugs and ward supplies	222 040	32.2
Other	58 468	8.5
**Total**	**687 939**	**100**

For patients ≤15, the average cost of treating one episode of cholera with recommended dosage of ORS, IVs, zinc supplement and antibiotics was US$14.49 (with erythromycin), US$14.77 (with ciprofloxacin) and US$18.03 (with azithromycin suspension). For patients ≥ 15 years old, the cost of treatment was US$17.66 (with ciprofloxacin) and US$35.16 (with azithromycin tablet). These costs of treatment were based on an average length of hospital stay of three days.

### Cost-effectiveness analysis

Of the 598,131 doses purchased and delivered locally at an estimated cost of US$1.72 per dose (US$1.31 and US$0.41 for purchase and local delivery cost of per dose, respectively), 424,100 were administered against an estimated target population of 578,043. Vaccine coverage was therefore estimated to be 73.4% [26]. Assuming a vaccine effectiveness of 63%, more (317) doses of vaccines would be required to avert a case of cholera as compared to 225 doses at a higher vaccine effectiveness of 89%. Similarly, the number of doses required to avert a case of death due to cholera was estimated at 16,708 doses at a lower vaccine effectiveness of 63% as compared to 11,841 doses at 89%. However, more cases and deaths (1,885) would be averted at 89% vaccine effectiveness as compared to 1,335 cases and deaths at 63%. Whereas only 706 DALYs would be averted on the basis of 63% vaccine effectiveness, 996 would be averted at 89%.

Supposing a life expectancy of 62 years and only one campaign every two years [40], the cost of a mass vaccination campaign with a single dose of Shancol was estimated at US$729,452 minus local delivery costs. When compared to the country’s GDP per capita threshold of US$1,500, the cost per DALY averted was less and estimated US$556–US$764 for patients ≥15 years old and US$516–US$765 for patients ≥15 years old. Computed costs per case averted were US$369–US$532 for patients ≥15 years old and US$352–US$528 for patients ≥15 years old. Table 7 shows the results of the CEA of the 2016 vaccine delivery campaign.

**Table 7:** Base-case estimates for cost-effectiveness of vaccination with single dose of Shancol.

Cost of vaccination	89% Vaccine Effectiveness				63% Vaccine Effectiveness			
	Patients ≤15 Years old		Patients ≥15 Years Old		Patients ≤15 Years old		Patients ≥15 Years Old	
Cases and deaths averted*^a^	1885		1885		1336		1336	
DALYs averted*^a^	996		996		706		706	
Doses per case averted*^a^	225		225		317		317	
Doses per death averted*^a^	11 841		11 841		16 708		16 708	
Total cost*^b^	729,452		729,452		729,452		729,452	
**Cost of treatment**	US$14.49^a^	US$18.03^b^	US$17.66^c^	US$35.16^d^	US$14.49^a^	US$18.03^b^	US$17.66^c^	US$35.16^d^
Cost averted	27 315	33 989	33 291	66 281	19 357	24 087	23 592	46 971
Net cost	702 137	695 463	696 1601	663 171	710 095	705 365	705 860	682 481
Cost per case averted	372	369	369	352	532	528	529	511
Cost per life saved	19 603	19 417	19 436	18 515	27 976	27 790	27 809	26 888
Total cost per DALY averted	705	698	699	666	1 006	1 000	1 000	967

*^a^ Total cases averted, DALYs averted and doses required to avert a single case of and death due to cholera are different across vaccine effectiveness but the same across ages distribution

*^b^ Total cost of vaccination campaign was the same across age distribution and vaccine effectiveness

^a,b^ Cost of treatment with Erythromycin and Azythromycin suspension for patients ≤15 years old

^c,d^ Cost of treatment with Ciprofloxacin and Azythromycin tablets for patients ≥15 years old

## Discussion

While some studies report vaccine delivery costs disaggregated by activities, several others disaggregate costs by inputs such as vaccine purchase, shipment, insurance, cold chain management, personnel incentives, training, transportation, social mobilization and local delivery. The delivery costs of OCVs via mass campaigns differ by country and even within the same country and the same setting [20,41–45]. The variations in cost estimates could considerably impact the scale up of OCV campaigns to prevent cholera outbreaks, hence the need to ascertain associated costs for individual settings.

In 2016, a study evaluated the feasibility of conducting a large-scale reactive cholera vaccination campaign in high-risk peri-urban areas of Lusaka and showed that a single dose of Shancol was purchased at US$1.31 and delivered to the community at US$0.41 per vaccine [26]. Therefore, about US$1.72 was spent per vaccinated person, 80% of which was attributed to vaccine purchase. In Hue City, Vietnam, the cost per vaccinated person was lower and estimated at US$1.07 with a higher proportion (90%) of the cost being spent on purchasing the vaccine [46]. Even though only a single dose was administered to 73.4% of targeted persons, the total cost of implementing the OCV campaign was somewhat high and estimated at US$1,152,291, including costs for local delivery for 578,043 doses. The total cost of implementing an OCV campaign could increase supposing all eligible residents in a cholera prone area were vaccinated with recommended doses of OCV. However, integrating mass vaccinations into the health system and conducting OCV campaigns routinely could marginally reduce costs [19].

Studies have reported higher incidence of cholera in households whose water source was not potable or did not have an in-house stand pipe [33]. In this study, about 75% of households which recorded a cholera case obtained their water from a public piped water source. The costs of improving WASH were not collected and can therefore not be compared with the costs of implementing OCVs. However, although cholera vaccination benefits individuals with potential herd immunity for the larger community, relevant authorities should consider prioritising surveillance and improving water supply and quality to prevent future cholera outbreaks [18].

In Zambia, cholera treatment is provided free of charge in public health facilities. Consequently, a large proportion of the total expenditure amounting to US$687,939 was borne by the health system and attributed to the purchase of medical supplies such as disinfectants, drugs, laboratory and ward supplies. This is consistent with other studies that reported that health care provider costs account for the largest proportion of costs [33,45]. However, in cases where patients had incurred costs, a large proportion of them were attributed to out-of-pocket costs and productivity losses [1]. Transport, communication and food and beverage costs were the major drivers for household costs contrary to other reports which attributed the highest costs to treatment of cholera [47]. The high expenditure on transport was attributable to distance and travel time from a patient’s house to the CTC [48,49].

Based on prescribed guidelines for treating cholera patients of different age groups, this paper considers treatment costs and vaccine effectiveness as key variables for calculating ICERs. Other studies have disregarded vaccine efficacy and demonstrated cost-effectiveness of OCV in endemic settings using cholera incidence and cost of vaccine as key variables [50].

Since the cost of treating cholera patients was based on two age groups, treatment costs per hospitalised patient with an episode of cholera were estimated US$14.77–US$ 18.03 for children ≤15 years old as compared to US$17.66– US$35.16 for older patients. In this study, the costs were highest for episodes treated with recommended doses of rehydration solutions, IV fluids, zinc supplement and erythromycin antibiotic. Corresponding costs for treating adults were similar in Beira, Mozambique (US$18.80) and Kolkata, India (US$17.90) but much higher in North Jakarta, Indonesia (US$134.00) [35]. Other studies have applied an average cost of treating hospitalized cases to all age groups [1]. Assuming two different vaccine effectiveness rates, the derived treatment costs were used to calculate the total cost per DALY averted for the two age groups.

This study demonstrates that vaccinating a single homogeneous population at risk of cholera disease is cost effective. These results are contrary to findings that suggest that vaccination programs that include adults can generally be less cost-effective than interventions targeted at all eligible children [18]. The costs per case averted during the 2016 OCV campaign were higher and estimated at US$369–US$532 for children ≤15 years old as compared to US$352–US$528 for older patients. This was similar to findings documented by Khan et al., 2018 that showed that varying costs of treatment do not substantially change the resulting ICERs [50].

Costs per case averted declined from US$699–US$666 for patients ≥15 years old and US$705-US$698 for patients ≤15 particularly when a higher vaccine effectiveness of 89% was used as a parameter to compute cost-effectiveness. Assuming a lower vaccine effectiveness of 63%, the total cost per DALY averted increased with a decrease in the cost of treatment. A study conducted in endemic settings demonstrated that the cost per case averted and per hospitalization averted declines with increasing cholera incidence, hence a very inexpensive vaccine becomes cost-effective only when incidence exceeds 1/1000 [51].

The results presented in this paper are unique because they include estimates of vaccine delivery and campaign costs, out-of-pocket and health systems costs and cost-effectiveness analysis of the vaccination campaigns with a single dose of Shancol a in peri-urban setting. Data from other studies were skewed towards adult hospitalised men and did not collect costs related to productivity losses for patients who had suffered cholera disease [33,45].

## Limitations

Outbreak driven diseases often have considerable costs that may not be associated with providing patient care. Such costs may be problematic to collect. For instance, this study did not calculate costs associated with loss of tourism due to the cholera outbreak or movement of economic activity from a cholera affected area to a non- affected area. Neither did we estimate time and money incurred by households on acquiring vaccines, loss of income due to stigma suffered by cholera patients nor costs of burying deceased cholera patients.

In addition, the analysis did not estimate health care provider costs associated with shifting of health funds and staff from routine services to outbreak management. The data presented in this paper may have been affected by patients’ recall bias since they were collected almost four months after patients had been treated for cholera.

The VICE calculator assumes that vaccines were administered to different groups with different characteristics. The tool allows users to divide populations into up to four groups (but total distribution should be 100%) or use a whole proportion. The proportions provided across each age category will be added together to get the resulting Total DALYs Averted. For this study, however, we were not able to divide the recipients of the OCV into groups as ages for the total number of people vaccinated was not available from the district health office. We therefore used one proportion (100%) for all age groups, hence the same DALYs averted in both groups. Drug costs are not based on the same sample of patients who were identified to provide costs incurred during cholera disease.

Lastly, the CFR is at least three times as high as in the overall outbreak and therefore suggests that the patients interviewed could have been sicker than the average patient during the outbreak, and likely incurred higher costs. This could have led to an overestimation of the CE of the vaccine.

## Conclusions

In conclusion, a cost-effectiveness analysis based on defined vaccine effectiveness and treatment costs shows that a vaccination campaign using a single dose of Shancol among a single, homogeneous population is very cost effective. However, policy makers must consider other budgetary and logistical factors such as vaccine purchase and delivery, personnel training, distribution of Information, Communication and Education (IEC) materials before deciding to introduce OCVs as a control measure for cholera. Since this CEA was based on a single dose OCV, costs of may be significantly higher if two doses were to be administered within recommended time intervals.

## Supporting information

S1 Appendix Cholera_Survey_Data_092016. (XLSX)

S2 Appendix Hospital and Health Facility Costs of Treating Cholera. (DOCX)

S3 Appendix Household questionnaire. (DOCX)

## References

[1] Sarker AR, Islam Z, Khan IA, Saha A, Chowdhury F, Khan AI, et al Cost of illness in a high risk urban area in Bangladesh: an analysis from household perspective. BMC Infectious Diseases. 2013; 13: 518 10.1186/1471-2334-13-518 24188717 PMC4228304

[2] Schaetti C, Weiss GM, Ali MS, Chaignat C, Khatib MA, Reyburn R, et al Cost of illness due to cholera, costs of immunisation and cost-effectiveness of an oral cholera mass vaccination campaign in Zanzibar. PLoS: Neglected Tropical Diseases. 2012; 6(10): e1844 10.1371/journal.pntd.0001844 23056660 PMC3464297

[3] World Health Organisation. WHO position paper—August 2017. 2017. [Internet] http://41.77.4.165:6510/apps.who.int/iris/bitstream/10665/258764/1/WER9234-477-498.pdf. Accessed 28 March 2018.

[4] World Health Organisation. Cholera Fact sheet. Geneva: World Health Organisation 2016 [Internet]. http://www.who.int/gho/epidemic_diseases/cholera/cases/en/. Accessed 30 Amrch 2018.

[5] Olu O, Babaniyi O, Songolo P, Matapo B, Chizema E, Kapin’a-Kanyanga M, Cholera epidemiology in Zambia from 2000 to 2010: implications for improving cholera prevention and control strategies in the country. East African Medical Journal. 2013; 90(10): 324–331. 26862642

[6] Siziya S. A review of the epidemic-prone enteric diseases in Zambia: cholera, typhoid fever and bacterial dysentery. The Health Press. 2017; 2(1): 6–11

[7] DuBois AE, Sinkala M, Kalurru P, Makasa-Chipoya M & Quick RE. Epidemic cholera in urban Zambia: hand soap and dried fish as protective factors. Cambridge University Press. 2006; 134: 1226–1230. 10.1017/S0950268806006273 16623992 PMC2870514

[8] Ali M, Nelson AR, Lopez AL, Sack DA. Updated global burden of cholera in endemic countries. PLoS: Neglected Tropical Diseases. 2015; 9(6). 10.1371/journal.pntd.0003832 26043000 PMC4455997

[9] Sinyange N, Brunkard MJ, Kapata N, Mazaba ML, Musonda GK, Hamoonga R, et al Cholera epidemic—Lusaka, Zambia., October 2017-May 2018. Morbidity and Mortality Weekly Report. 2018; 67(19). 10.15585/mmwr.mm6719a5 PMC6048949 29771877

[10] Kimball AM, Wong KY, Taneda K. An evidence base for international health regulations: quantitative measurement of the impacts of epidemic disease on international trade. Revue Scientifique et Technique. 2005; 24: 825–832. 16642753

[11] Kirigia JM, Sambo LG, Yokouide A, Soumbey EA, Muthuri LK, & Kirigia DG. Economic burden of cholera in the WHO African region. BMC International Health and Human Right. 2009; 9(8). 10.1186/1472-698X-9-8 19405948 PMC2691726

[12] Taylor DL, Kawahita MT, Cairncross S, & Ensink JH. Tha. Impact of water, sanitation and hygiene interventions to control cholera: A systematic review. PLoS: One. 2015; 10(8): e0135676 10.1371/journal.pone.0135676 26284367 PMC4540465

[13] Yates T, Allen JV, Joseph ML, & Lantagne D. Wash interventions in disease outbreak response: Humanitarian Innovation and Evidence Program. Oxfam GB. 2017; http://fic.tufts.edu/assets/WASH-Systematic-Review.pdf.

[14] Deen J, Ali M, Sack D. Methods to assess the impact of mass Oral Cholera Vaccination campaigns under real field conditions. PLoS One: 9(2): e88139 10.1371/journal.pone.0088139 24516595 PMC3917865

[15] World Health Organisation. WHO Prequalified Vaccines. Geneva, Switzerland: WHO 2016.

[16] Longini IM Jr., Nizam A, Ali M, Yunus M, Shenvi N, et al Controlling endemic cholera with oral vaccines. PLoS: Medicine. 2007; 4(11): 1776–1783. 10.1371/journal.pmed.0040336 18044983 PMC2082648

[17] Sack DA. Herd protection and herd amplification in cholera. Journal of Health Population Nutrition. 2006; 24:1–5. 16796143

[18] Jeuland M, Cook J, Poulos C, Clemens J, & Whittington D. Cost-effectiveness of new-generation oral cholera vaccines: a multisite analysis. International Society for Pharmacoeconomics and Outcomes Research. 2009; 12(6): 899–908 10.1111/j.1524-4733.2009.00562.x 19824189

[19] Cavailler P, Lucas M, Perroud V, McChesney M, Ampuero S, Guerin JP, et al Feasibility of a mass vaccination campaign using a two-dose oral cholera vaccine in an urban cholera-endemic setting in Mozambique. ScienceDirect. 2005; 24(2006): 4890–4895. 10.1016/j.vaccine.2005.10.006 16298025

[20] Ciglenecki I, Sakoba K, Luquero JF, Heile M, Itama C, et al Feasibility of mass vaccination campaign with oral cholera vaccines in response to an outbreak in Guinea. PLoS Medicine. 2013; 10(9). 10.1371/journal.pmed.1001512 24058301 PMC3769208

[21] Desai NS, Pezzoli L, Martin S, Costa A, Rodriguez C, Legros D, et al A second affordable oral cholera vaccine: implications for the global vaccine stockpile. The Lancet. 2016; 4(4). 10.1016/S2214-109X(16)00037-1 27013303

[22] Ilboudo PG and Le Gargasson JB. Delivery cost analysis of a reactive mass cholera vaccination campaign: a case study of Shancol vaccine use in Lake Chilwa, Malawi. BMC Infectious Diseases. 2017; 17(1): 779 10.1186/s12879-017-2885-8 29258447 PMC5735524

[23] Kim SY, Choi Y, Mason PR, Rusakaniko S, Goldie SJ. Potential impact of reactive vaccination in controlling cholera outbreaks: an exploratory analysis using a Zimbabwean experience. South African Medical Journal. 2011; 101(9): 659–656. 21920160

[24] Khan AI, Levin A, Chao DL, DeRoeck D, Dimitrov DT, Khan JAM, et al The impact and cost-effectiveness of controlling cholera through the use of oral cholera vaccines in urban Bangladesh: A disease modeling and economic analysis. PLoS Neglected Tropical Disease. 2018; 12(10): e0006652 10.1371/journal.pntd.0006652 PMC6177119 30300420

[25] Central Statistics Office. 2010 Census of population and housing: Lusaka province analytical report. 2014.

[26] Poncin M, Zulu G, Vute C, Ferreras E, Muleya-Mbwili C, Malama K, et al Implementation research: mass vaccination with single-dose oral cholera vaccine, Zambia. World Health Organisation Bulletin. 2018; 96(2): 86–93. 10.2471/BLT.16.189241. PMC5791774 29403111

[27] Parker LA, Rumunu J, Jamet C, Kenyi Y, Lino RL, Wamala JF et al Neighborhood-targeted and case-triggered use of a single dose of oral cholera vaccine in an urban setting: Feasibility and vaccine storage. PLoS Neglected Tropical Diseases. 2017 11(6). [10.137/journal.pntd.0005652]. 10.1371/journal.pntd.0005652 PMC5478158 28594891

[28] World Health Organisation. Choosing interventions that are cost effective (WHO-CHOICE): Threshold values for intervention cost-effectiveness by region. Geneva: WHO 2008 [Internet]. http://www.who.int/choice/en/. Accessed 15 February 2018.

[29] Medical Stores Limited. MSL Drug Catalogue. October 2016

[30] Troeger C, Chao D, Sack D. VICE. Centre for Statistical and Quantitative Infectious Disease (CSQUID), Fred Hutchinson Cancer Research Centre and The Dove project at Johns Hopkins Bloomberg School of Public Health. [Internet] https://www.stopcholera.org/toolkits/stopcholera-toolkit/vice-calculator. Accessed 9th April 2018.

[31] Ferreras E, Chizema-Kawesha E, Blake A, Chewe O, Mwaba J, Zulu G, et al Single-dose cholera vaccine in response to an outbreak in Zambia. New England Journal of Medicine. 2018; 378(6): 577–579. 10.1056/NEJMc1711583 29414267

[32] Ivers LC, Hilaire I, Teng JE, Almazor CP, Jerome JG et al Effectiveness of reactive oral cholera vaccination in rural Haiti: a case-control study. The Lancet Global Health. 2015; 3(3): e162–e168. https://1016/S2214-109X(14)70368-7 25701994 10.1016/S2214-109X(14)70368-7 PMC4384694

[33] Poulos C, Riewpaiboon A, Stewart JF, Clemens J, Guh S, et al Costs of illness due to endemic cholera. Epidemiology and Infection. 2011; 140:1–10. 10.1017/S095026881000316X 21554781 PMC3824392

[34] The World Bank. https://data.worldbank.org/inidcator/sp.dyn.le00.in)

[35] Macroeconomics and health: investing in health for economic development. Report for the Commission on Macroeconomics and Health. 2001. Geneva. WHO.

[36] Salomon JA, Vos T, Hogan DR, Gagon M, Naghavi M, Mokdad A et al Coomon values in assessing health outcomes from disease and injury: Disability weights measurement study for the global burden of disease study. The Lancet. 2010. 2012; 380(9859):2129–2143. 10.1016/S0140-6736(12)61680-8 PMC10782811 23245605

[37] Rice DP. Estimating the cost of illness. American Journal of Public Health. 1967; 57(3) 10.2105/ajph.57.3.424 PMC1227175 6066903

[38] IVI, WHO, DOVE. (2016). CHOLTOOL: Planning and costing. User’s Guide, Seoul, South Korea. In. Seoul, South Korea.

[39] Morgan W, Levin A, Hutubessy RC, Mogasale V. Costing oral cholera vaccine delivery using a generic oral cholera vaccine delivery planning and costing tool (CHOLTOOL). International Vaccine Institute 2017. 10.1080/21645515.2020.1747930 PMC8641596 32530361

[40] Azman AS, Luquero FJ, Ciglenecki I, et al The Impact of a One-Dose versus Two-Dose Oral Cholera Vaccine Regimen in Outbreak Settings: A Modeling Study. PLoS Medicine 2015; 12: 1–18. 10.1371/journal.pmed.1001867 PMC4549326 26305226

[41] Khan IA, Saha A, Chowdhury F, Khan AI, Uddin MJ, Begum YA, Riaz BK, Islam S, Ali M, Luby SP, et al Coverage and cost of a large oral cholera vaccination program in a high-risk cholera endemic urban population in Dhaka, Bangladesh. Vaccine. 2013;31(51):6058–64. 10.1016/j.vaccine.2013.10.021 24161413

[42] Mogasale V, Ramasani E, Wee H, Kim JH. Oral Cholera Vaccination delivery cost in low-and-middle income countries: An analysis based on systematic review. PloS Neglected Tropical Diseases. 2016; 10(12): e0005124 10.1371/journal.pntd.0005124 27930668 PMC5145138

[43] Kar KS, Sah B, Patnaik B, Kim HY, Kerketta SA, Shin S et al Mass vaccination with a new, less expensive Oral Cholera Vaccine using public health infrastructure in India: the odisha model. PloS: Neglected Tropical Diseases. 2014; 8(2): 10.1371/journal.pntd.0002629 24516675 PMC3916257

[44] Teshome S, Desai S, Kim JH, Belay D, Mogasale V. Feasibility and costs of a targeted cholera vaccination campaign in Ethiopia. Human Vaccines & Immunotherapeutics. 2018 10.1080/21645515.2018.1460295 PMC6290934 29648523

[45] Ilboudo PG, Huang XX, Ngwira B, Mwanyungwe A, Mogasale V, Mengel MA. Cost-of-illness of cholera to households and health facilities in rural Malawi. PloS: One. 2017; 12(9). 10.1371/journal.pone.0185041 28934285 PMC5608291

[46] Anh DD, Lopez AL, Tran HTM, Cuong NV, Thiem VD, Ali M, et al Oral Cholera Vaccine development and use in Vietnam. PLoS Medicine. 2014; 11(9): e1001712 10.1371/journal.pmed.1001712 25180511 PMC4151976

[47] Awalime KD, Davies-Teye KBB, Vanotoo AL, Owoo SN, Nkhetiah-Ampo. Economic evaluation of 2014 cholera outbreak in Ghana: A household cost analysis. Health Economics Review. 2017; 7(45). 10.1186/s13561-017-0182-2 29204727 PMC5714944

[48] Whittington D, Radin M, Jeuland M. Economic costs and benefits of cholera vaccination intervention options for Haiti. Copenhagen Consensus Centre 2017.

[49] Qadri F, Ali M, Chowdhury F, et al Feasibility and effectiveness of oral cholera vaccine in an urban endemic setting in Bangladesh: A cluster randomised open-label trial. Lancet 2015; 386: 1362–1371. 10.1016/S0140-6736(15)61140-0 26164097

[50] Khan AI, Levin A, Chao DL, DeRoeck D, Dimitrov DT, Khan JAM, et al The impact and cost-effectiveness of controlling cholera through the use of oral cholera vaccines in urban Bangladesh: A disease modeling and economic analysis. PLoS Neglected Tropical Disease. 2018 12(10): e0006652 10.1371/journal.pntd.0006652 PMC6177119 30300420

[51] Chaignat CL and Monti V. Use of cholera vaccine in complex emergencies: What next? Summary report of expert meeting and recommendations of WHO. BMC: Journal of Health, Population and Nutrition. 2007; 25(2). PMC2753992 17985828

