# Supplementary material for: Evaluating the costs of cholera illness and cost-effectiveness of a single dose oral vaccination campaign in Lusaka, Zambia
Source: PLoS One. 2019 May 31;14(5):e0215972. doi: 10.1371/journal.pone.0215972 (PMC6544210; doi:10.1371/journal.pone.0215972)
Supplement: S2 Appendix — (DOCX) [file pone.0215972.s002.docx]

**Costing and Cost Effectiveness Analysis of the Oral Cholera Vaccine Campaign in Lusaka, Zambia in 2016:**

**Questionnaire for Collecting Costs of Treating Cholera at a Health Facility**

Table of Contents

[Section 1: Introduction and Consent 3](#_Toc460498333)

[Section 2: General Facility Information 4](#_Toc460498334)

[Section A: Identification of facility 4](#_Toc460498335)

[Section B: Identification of respondent 4](#_Toc460498336)

[Section C: Statistics on cholera cases 4](#_Toc460498337)

[Section D: Health personnel time costs 5](#_Toc460498338)

[Section E: Costs of health personnel 8](#_Toc460498339)

## Module 1: Introduction and Consent

Hello, my name is ____________________________________ [*introduce all staff present*]. We are working with the Ministry of Health to collect information about the costs of treating the cholera episode. The purpose of this interview is to obtain costs borne by your health facility during a cholera episode. We expect to spend 1 hour in your facility. Before you decide to take part in this study, we would like to explain the purpose of the study, any risks and benefits to you and what is expected of you.

Taking part in this survey is voluntary and the information you provide will be kept strictly confidential and none of your names will appear in any report. The information will be used only for the costing and cost-effectiveness study with the aim of calculating costs associated with treating cholera episodes. Feel free to ask for any clarification on unclear issues during this interview. Choosing not to participate will not involve any penalty, but the information you provide will enable us estimate costs to prevent and handle future cholera episodes. You may choos to withdraw from the assessment anytime during the interview without penalty to you or your facility.

If you have any questions or concerns about this assessment, please feel free to contact ________________________________ [*provide name of designated costing contact*] and telephone number [*provide telephone number*] ­­­­­­­­­­­­­­­­­­­­___________________________________.

**Consent to Participate:**

I have read [heard] the information provided above and i understand it. I have been allowed to ask questions and all of my questions have been answered to my satisfaction.

**_____________________________ ____________________________**

**Name of Interviewee Phone Number of Interviewee**

**_____________________________ / /**

**Signature of Interviewee** Date (dd/mm/yy)

**_____________________________ ___________________________**

**Name of Interviewer Phone Number of Interviewer**

**_____________________________ / /**

**Signature of Interviewer** Date (dd/mm/yy)

## Module 2: General Facility Information

This questionnaire is to be completed by interviewing health care providers in health facilities with Cholera Treatment Centres (CTCs). Interviewees must have been in contact and involved with providing treatment and care for cholera patients during the most recent cholera outbreak.

### Section A: Identification of facility

A1. Date of the interview: /  **/**

Date (dd/mm/yy)

A2. Health facility name: ___________________________________________________

A3. Province:­­­­­­­­­­­­­­­­­­­­___________________________ A4. Provincial Code:

A5. District:____________________________ A6. District Code:

### Section B: Identification of respondent

B1. First and last names of respondent:_________________________________________

B2. Title of respondent:_____________________________________________________

B3. Contact number of respondent:____________________________________________

### Section C: Statistics on cholera cases

C1. Have you recorded the total number of people treated for cholera in this health facility during the recent outbreak?

Yes (1) No (2)🡪**Go to C3** Do not know (97979) 🡪**Go to C3**

C2. How many **cases of cholera** have been treated in this health facility **during the last 12 months**? (Please consult the cholera treatment register or other official records at the health facility to verify number of cholera cases)

(Do not know=97979) 🡪**Go to C3**

C3. How many of the cholera cases treated at this health facility were admitted for 1-3 days??

(Do not know=97979)

C4. How many of the cholera cases treated at this health facility, were admitted for 3-7 days?

(Do not know=97979)

C5. How many of the cholera cases treated at this health facility were admitted for more than 7 days?

(Do not know=97979)

*Interviewer make sure that the total of C3+C4+C5 equals C2, if not please ask the respondent to amend numbers.*

Read out: It is important for this study to have a good estimate of the distribution of cholera cases treated. The following questions ask you to think about 10 patients treated for cholera.

### Section D: Health personnel time costs

***Now, I would like you to only think about 10 outpatient cases treated for cholera in your structure.***

D1. Of the **10 patients** treated for cholera in your health facility, how many were seen/cared for by each of the **staff listed below**. Please give your best expert estimate of the time spent by **each staff on a single patient** from **admission to discharge.**

| **N°** | **Category of health worker** | **Did this health worker provided care to the patient?**  **Yes=1 ; No=0** | **Estimated number of patients cared for out of 10 cholera patients.** | **Estimated time spent between admission and discharge (in minutes) to treat a single patient** |
| --- | --- | --- | --- | --- |
| 1 | Doctor (Medical Officer) |  |  |  |
| 2 | Medical Licentiate |  |  |  |
| 3 | Clinical Officer |  |  |  |
| 4 | Nurse/Midwife |  |  |  |
| 5 | Environmental Health Technician |  |  |  |
| 6 | Pharmacist |  |  |  |
| 7 | Laboratory Technologist |  |  |  |
| 8 | Community Health Worker |  |  |  |
| 9 | Other (Specify)  ………………………... |  |  |  |

***Now, I would like you to only think about 10 inpatient cases that spent 1-3 days to treat cholera in your health facility.***

D2. Among **these 10 patients**, how many were seen/cared for by each of the **staff listed below**. Please give your best expert estimate of the time spent by **each staff on a single patient** from **admission to discharge.** Please remember these patients spent between 1and 3 days in the health facility. Time estimate should therefore cover the period from admission to discharge and presented in terms of minutes (**Skip to D3 if C6=0**)

| **N°** | **Category of health worker** | **Did this health worker provided care to the patient?**  **Yes=1 ; No=0** | **Estimated number of patients cared for out of 10 cholera patients.** | **Estimated time spent between admission and discharge (in minutes) to treat a single patient** |
| --- | --- | --- | --- | --- |
| 1 | Doctor (Medical Officer) |  |  |  |
| 2 | Medical Licentiate |  |  |  |
| 3 | Clinical Officer |  |  |  |
| 4 | Nurse/Midwife |  |  |  |
| 5 | Environmental Health Technician |  |  |  |
| 6 | Pharmacist |  |  |  |
| 7 | Laboratory Technologist |  |  |  |
| 8 | Community Health Worker |  |  |  |
| 9 | Other (Specify)  ………………………... |  |  |  |

***Now, I would like you to only think about 10 inpatient cases that spent more than 7 days to treat cholera in your health facility.***

D3. Among **these 10 patients**, how many were seen/cared for by each of the **staff listed below**. Please give your best expert estimate of the time spent by **each staff on a single patient** from **admission to discharge.** Please remember these patients spent more than 7 days in the health facility. Time estimate should therefore cover the period from admission to discharge and presented in terms of minutes (**Skip to D4 if C7=0**)

| **N°** | **Category of health worker** | **Did this health worker provided care to the patient?**  **Yes=1 ; No=0** | **Estimated number of patients cared for out of 10 cholera patients.** | **Estimated time spent between admission and discharge (in minutes) to treat a single patient** |
| --- | --- | --- | --- | --- |
| 1 | Doctor (Medical Officer) |  |  |  |
| 2 | Medical Licentiate |  |  |  |
| 3 | Clinical Officer |  |  |  |
| 4 | Nurse/Midwife |  |  |  |
| 5 | Environmental Health Technician |  |  |  |
| 6 | Pharmacist |  |  |  |
| 7 | Laboratory Technologist |  |  |  |
| 8 | Community Health Worker |  |  |  |
| 9 | Other (Specify)  ………………………... |  |  |  |

***Now, I would like you to only think about 10 inpatient cases that spent 3-7 days to treat tcholera in your health facility.***

D4. Among **these 10 inpatient cases**, how many were seen/cared for by each of the **staff listed below**. Please give your best expert estimate of the time spent by **each staff on a single patient** from **admission to discharge.** Please remember these patients spent between 3 and 7 days in the health facility. Time estimate should therefore cover the period from admission to discharge and presented in terms of minutes (**Skip to D5 if C8=0**)

| **N°** | **Category of health worker** | **Did this health worker provided care to the patient?**  **Yes=1 ; No=0** | **Estimated number of patients cared for out of 10 cholera patients.** | **Estimated time spent between admission and discharge (in minutes) to treat a single patient** |
| --- | --- | --- | --- | --- |
| 1 | Doctor (Medical Officer) |  |  |  |
| 2 | Medical Licentiate |  |  |  |
| 3 | Clinical Officer |  |  |  |
| 4 | Nurse/Midwife |  |  |  |
| 5 | Environmental Health Technician |  |  |  |
| 6 | Pharmacist |  |  |  |
| 7 | Laboratory Technologist |  |  |  |
| 8 | Community Health Worker |  |  |  |
| 9 | Other (Specify) |  |  |  |

D5. Do people sick with cholera pay for hospitalization?

Yes (1) No (2)🡪**Go to E**

D5. How much do they pay per day?

(Don’t know=979797)

### Section E: Costs of health personnel

E1. Is this health facility only dedicated to treating cholera cases only?

Yes (1) 🡪**Go to E5** No (2)

E2. How many patients have been treated for **all other diseases than cholera** in the health facility **during the last 12 months** ?

(Don’t know =979797)

E3. Out of the number given in **E2**, how many of them necessitated hospitalization?

(Don’t know =979797)

E4. What is the average length of hospitalization in this health facility ?

(Don’t know =979797)

*Now, I would like to ask you to give an estimate of* ***capital (fixed) costs****. It is difficult to have a precise figure of capital* ***costs*** *and so we will ask you to give the best estimate possible.*

*Note: If the health facility is a Cholera Treatment Centre (CTC), please consult financial documents from health facility and/or district health office to get the full cost of setting up and equipping the CTC.*

E5. What is the estimate cost of building and equipping a health facility such as yours?

(Don’t know =979797)

E6. How long do you estimate the utility life years of the equipped health facility?

Years

(Don’t know =979797)

E7. What is the estimated salary for each of the categories of health workers indicated?

| **N°** | **Category of Staff** | **Gross Wage**  **(ZMK)** |
| --- | --- | --- |
| 1 | Doctor (Medical Officer) |  |
| 2 | Medical Licentiate |  |
| 3 | Clinical Officer |  |
| 4 | Nurse/Midwife |  |
| 5 | Environmental Health Technician |  |
| 6 | Pharmacist |  |
| 7 | Laboratory Technologist |  |
| 8 | Community Health Worker |  |
| 9 | Other (Specify) …………………… |  |

Now, I would like to ask you to report on costs incurred on running this facility in the last 12 months. Please include cost for non-medical staff (administrative staff).

E8. How much has been spent on each of the cost items listed?

| **N°** | **Expenses for running the facility** | **Costs in Kwacha** |
| --- | --- | --- |
| 1 | Maintenance of buildings |  |
| 2 | Maintenance of equipments |  |
| 3 | Maintenance of rolling stock |  |
| 4 | Maintenance of computers |  |
| 5 | Purchase of drugs and medical supplies |  |
| 6 | Transportation including fuel and hire of vehicle |  |
| 7 | Accomodation |  |
| 8 | Subsistence allowance |  |
| 9 | Furnitures |  |
| 10 | Electricity |  |
| 11 | Telephone |  |
| 12 | Gaz |  |
| 14 | Cost of administrative staff clinical staff excluded |  |
| 15 | Training and scholarships |  |
| 16 | Training venue |  |
| 17 | Training materials |  |
| 18 | Other (Specify)……………………………… |  |

## Module 3 : Costs of Treating Cholera

This section will collect informagtion about costs incurred by your health facility to treat patients who suffered from cholera. (Please review patient clinical files for detailed information).

### Section A : Identification of Patient

A1. Date of admission : / /

dd / mm / yy

A2. Patient ID : - - -

A3. Age of patient :

A4. Patient’s sex : Male=1 Female=0

### Section B: Drugs Used to Treat Cholera Patient

Please, specify for drugs used, quantity and unit cost. Use the empty cells to inform the medicines we have not listed and have been used.

| **Drug Name** | **Unit presentation of drug** | **Quantity used** | **Unit cost** |
| --- | --- | --- | --- |
| Oral rehydration salts | Bag |  |  |
| Ringers Lactate |  |  |  |
| Doxcycline |  |  |  |
| Azithromycin |  |  |  |
| Zinc supplementation |  |  |  |
| Other (Specify)  .................................................... |  |  |  |
| Other (Specify)  .................................................... |  |  |  |
| Other (Specify)  .................................................... |  |  |  |
| Other (Specify)  .................................................... |  |  |  |
| Other (Specify)  .................................................... |  |  |  |

### Section C : Materials and Consumables Used

Fill each consumable material, quantity and its unit cost. Use the empty cells to report information about consumables not listed.

| **Consumable** | **Unit presentation** | **Quantity** | **Unit cost** |
| --- | --- | --- | --- |
| Care book | Unit |  |  |
| Vaccination card |  |  |  |
| Clinical file |  |  |  |
| Examination gloves |  |  |  |
| Cannulla G24 |  |  |  |
| Sterile collection pot |  |  |  |
| Seringe 10 cc + needle |  |  |  |
| Seringe 5cc+needle |  |  |  |
| Stool sample test |  |  |  |
| Crystal VC dipstick |  |  |  |
| Other (Specify)  .................................................... |  |  |  |
| Other (Specify)  .................................................... |  |  |  |
| Other (Specify)  .................................................... |  |  |  |
| Other (Specify)  .................................................... |  |  |  |
| Other (Specify)  .................................................... |  |  |  |

Observations:................................................................................................................................

.......................................................................................................................................................

.......................................................................................................................................................

........................................................................................................................................................

We have come to the end of this interview. Thank you for your time!
