# Supplementary material for: Evaluating the costs of cholera illness and cost-effectiveness of a single dose oral vaccination campaign in Lusaka, Zambia
Source: PLoS One. 2019 May 31;14(5):e0215972. doi: 10.1371/journal.pone.0215972 (PMC6544210; doi:10.1371/journal.pone.0215972)
Supplement: S3 Appendix — (DOCX) [file pone.0215972.s003.docx]

**Costing and Cost Effectiveness Analysis of the Oral Cholera Vaccine Campaign in Lusaka, Zambia in 2016:**

**Household Questionnaire**

**Cost of Cholera Disease Study**

## Module 1: Introduction and Consent

Hello, my name is ____________________________________ [*introduce all staff present*]. We are working with the Ministry of Health to collect information about the costs of cholera disease. The purpose of this interview is to obtain costs borne by your household during a cholera episode by a member of your household. We expect to spend 1 hour with you. Before you decide to take part in this study, we would like to explain the purpose of the study, any risks and benefits to you and what is expected of you.

Taking part in this survey is voluntary and the information you provide will be kept strictly confidential and none of your names will appear in any publication. The information will be used only for the costing and cost-effectiveness study with the aim of calculating costs incurred by your household during the cholera episode. Feel free to ask for any clarification on unclear issues during this interview. Choosing not to participate will not involve any penalty, but the information you provide will enable us estimate costs to prevent and handle future cholera episodes. You may choose to withdraw from the interview anytime during the interview without penalty to you or your household.

If you have any questions or concerns about this assessment, please feel free to contact ________________________________ [*provide name of designated costing contact*] and telephone number [*provide telephone number*] ­­­­­­­­­­­­­­­­­­­­___________________________________.

**Consent to Participate:**

I have read [heard] the information provided above and i understand it. I have been allowed to ask questions and all of my questions have been answered to my satisfaction.

**_____________________________ ____________________________**

**Name of Interviewee Phone Number of Interviewee**

**_____________________________ / /**

**Signature of Interviewee** Date (dd/mm/yy)

**Participant ID**

**_____________________________ ___________________________**

**Name of Interviewer Phone Number of Interviewer**

**_____________________________ / /**

**Signature of Interviewer** Date (dd/mm/yy)

# Module 2: General Information

|  |
| --- |

A0. Time at which interview started: ( HH/MN/SS)

## Section A: Identification of the participant

A1. Identification number in the study

(*Interviewers make sure to concisely report the ID number you gave on the consent form*)

|  |
| --- |

A2. Date of the interview: (DD/MM/YY)

A3. Where is the patient from?

Bauleni (1) Kanyama (2) Chawama (3)

Other, specify (4) …………………………………………

A4. Place where the interview took place:

Cholera Treatment Center (CTC) at public health facility (1)

Cholera Treatment Center (CTC) at private health facility (2)

Cholera treatment unit (CTU) at public health facility (3)

Public health facility (without CTC or CTU) (4)

Private health care facility (without CTC or CTU (5)

Other, Specify (6) …………………………………………

A5. Is the participant living in a rural or an urban area?

Rural area (1) Urban area (2)

**Interviewer: In conducting the interview, all darkened sections must be read out to the participant**

## Section B: Patient’s demographic characteristics

B1. What is the sex of the patient? Male (1) Female (2)

B2. How old were you at your last birthday?

(*Completed years*) (*Completed months*)

B3. Could you please tell me your relationship with the patient?

Self (patient) (1) Mother (2) Father (3)

Sister (4) Brother (5) Grandmother (6)

Grandfather (7) Other, specify (8) …………………………………………

B4. What is the highest level of schooling you (the patient) have attained?

Primary (1) Lower secondary (2)

Upper secondary (3) Tertiary (College/ University) (4)

Other, specify (5) …………………………………………

B5. What is your (the patient) religion?

Christian (1) Bahai Faith (2) Buddhist (3)

Islam (4) Hindu (5) Muslim (6)

Other, specify (7) …………………………………………

B8. What is your marital status?

Cohabiting (1) Divorced (2)

Married (3) Separated (4)

Widowed (5) Other, specify (6) …………………………………………

## Section C: Patient’s health history before treatment in this health facility

**First, I would like to know how much you (the patient) had to spend on any healthcare services (including traditional healers) against cholera**

C1. How many days were you (the patient) sick with diarrhea and vomiting before you were diagnosed with cholera?

Days *(don’t know=97)*

C2. Did you receive any treatment for the diarrhea and vomiting before you were diagnosed with cholera?

Yes (1)

No (2) **→ *Go to C6***

Do not know (3) **→ *Go to C6***

C3. Where else did you seek treatment for the diarrhea and vomiting before you were diagnosed with cholera? (Respondent can select more than one response)

District hospital (1) Health centre/post (2) Cholera treatment centre (3)

Cholera treatment unit (4) Traditional healer (5)

Other, specify (6) …………………………………………

*I would like to ask questions regarding the* ***first visit*** *you have done in seeking care for your cholera illness*

C4. Did you (or the patient) buy food and/or water for yoursel (the patient) during your visits?

|  | **Period of Visit** | | | | | |
| --- | --- | --- | --- | --- | --- | --- |
|  | 1^st^ Visit | 2^nd^ Visit | 3^rd^ Visit | 4^th^ Visit | 5^th^ Visit | 6^th^ Visit |
|  |  |  |  |  |  |  |
|  |  |  |  |  |  |  |
|  |  |  |  |  |  |  |
|  |  |  |  |  |  |  |

Did you (or the patient) buy food and/or water for yoursel (the patient) during your first visit?

Yes (1) No (2) **→ *Go to C6***

Don’t remember (3) **→ *Go to C6*** Don’t know (4) **→ *Go to C6***

C5. How much did you pay for the food and/or water during your first visit?

(*don’t know =979797)*

C6. Did you (or the patient) pay any consultation fee (to doctors, nurses, traditional healers) during the first visit?

Yes (1) No (2) **→ *Go to C8***

Don’t remember (3) **→ *Go to C8*** Don’t know (4) **→ *Go to C8***

C7. How much did you pay for consultations?

(*don’t know =979797)*

C8. Did you (or the patient) pay for any medicine/drugs during the first visit?

Yes (1) No (2) **→ *Go to C11***

Don’t remember (3) **→ *Go to C11*** Don’t know (4) **→ *Go to C11***

C9. How much did you pay for medicine/drugs (excluding herbs and roots from healers)?

(*don’t know=979797)*

C10. Did you (or the patient) pay any fees to traditional healers during the first visit,?

Yes (1) No (2) **→ *Go to C13***

Don’t remember (3) **→ *Go to C13*** Don’t know (4) **→ *Go to C13***

C11. How much did you pay for roots and herbs from healers (excluding modern medicine/drugs)?

(*don’t know=979797)*

C12. During the first visit, did you (or the patient) pay any diagnostic/lab fee?

Yes (1) No (2) **→ *Go to C15***

Don’t remember (3) **→ *Go to C15*** Don’t know (4) **→ *Go to C15***

C13. How much did you pay for diagnostic/lab tests?

(*don’t know=979797)*

C14. During the first visit, did you (or the patient) pay any diagnostic/lab fee ?

Yes (1) No (2) **→ *Go to C17***

Don’t remember (3) **→ *Go to C17*** Don’t know (4) **→ *Go to C17***

C15. How much did you pay for diagnostic/lab tests?

(*don’t know=979797)*

C16. During the first visit, did you (or the patient) pay any fee for transportation (from your

house to the first facility and back to your house)?

Yes (1) No (2) **→ *Go to C19***

Don’t remember (3) **→ *Go to C19*** Don’t know (4) **→ *Go to C19***

C17. How much did you pay for the round trip (forth and back to your house)?

(*don’t know=979797)*

C18. During the first visit, did you (or the patient) pay any fee for hospitalization?

Yes (1) No (2) **→ *Go to C21***

Don’t remember (3) **→ *Go to C21*** Don’t know (4) **→ *Go to C21***

C19. How much did you pay for hospitalization?

(*don’t know=979797)*

C20. During the first visit, did you (or the patient) pay any other fee outside what we have

mentioned above?

Yes (1) No (2) **→ *Go to C24***

Don’t remember (3) **→ *Go to C24*** Don’t know (4) **→ *Go to C24***

C21. Please specify what you paid for?

…………………………………………………………………………………..

C22. How much did it costs? (*don’t know=979797)*

C23. In case you (or the patient) do not remember costs borne during the first visit, can you estimate how much you spent during your first visit?

0-100 ZMW (1) 101-500 ZMW (2) 501-1,000 ZMW (3)

1,001-2,000 ZMW (4) over 2,000 ZMW (5)

C24. What was the main means of transportation you (or the patient) used during the first visit?

On foot/walking (1) Bicycle (2) Boat (3)

Motorcycle (4) Minibus (5) Private car (6) Horse cart (6) Other (8) Specify…………………………………

C25. How long did you (the patient) took to get to the place of your first visit (consider round trip)?

Minutes (*don’t know=979)*

C26. Excluding you (the patient), how many people in total accompanied you (the patient) to the place of the first visit?

Number 🡪 **if 00 or 97 Go to C33**

(*don’t know=97 ; 00 if no one accompanied the patient )*

C27. Out of the total in **C26**., how many people used a separate means of transportation (*people that did not travel simultaneously using the same means of transportation as you (the patient*)?

Number (*don’t know=97)*

C28. On average, how much did a round trip cost to each accompany person (*approximate please*)?

ZMK (*don’t know=979797 ; no expense=000000)*

C29. Excluding you (the patient), on average, how much did each accompany person spend on food/water

during your first visit (*approximate please*)?

ZMK (*don’t know=979797; no expense=000000)*

C30. Out of the total in **C28.**, how many accompany person booked lodging during your first visit? (*approximate please*)

Number (**don’t know=97 or if 00 🡪 Go to 35***)*

C31. How much did each accompany person spend on lodging during your first visit (*approximate please*)?

ZMK (*don’t know=979797; no expense=000000)*

*I would like to ask questions regarding the* ***second visit*** *you have done in seeking care for your cholera*

*illness*

C35. As to your memory, where did you seek treatment?

District hospital (1) Health centre/post (2)

Private facility (3) Traditional healer (4)

Cholera treatment centre (5) Cholera treatment unit (6)

Other , specify (7) …………………………………………………………..

C36. During the second visit, did you (or the patient) buy food and/or water for yourself

(the patient)?

Yes (1) No (2) **→ *Go to C38***

Don’t remember (3) **→ *Go to C38*** Don’t know (4) **→ *Go to C38***

C37. How much did you pay for the food and/or water during your second visit?

(*don’t know =979797)*

C38. During the second visit, did you (or the patient) pay any consultation fee (to doctors,

nurses, healers)?

Yes (1) No (2) **→ *Go to C40***

Don’t remember (3) **→ *Go to C40*** Don’t know (4) **→ *Go to C40***

C39. How much did you pay for consultations? (*don’t know =979797)*

C40. During the second visit, did you (or the patient) pay any medicine/drugs?

Yes (1) No (2) **→ *Go to C42***

Don’t remember (3) **→ *Go to C42*** Don’t know (4) **→ *Go to C42***

C41. How much did you pay for medicine/drugs (excluding herbs and roots from healers)?

(*don’t know=979797)*

C42. During the second visit, did you (or the patient) pay any fee to traditional healers?

Yes (1) No (2) **→ *Go to C44***

Don’t remember (3) **→ *Go to C44*** Don’t know (4) **→ *Go to C44***

C43. How much did you pay for roots and herbs from healers (excluding modern medicine/drugs)?

(*don’t know=979797)*

C44. During the second visit, did you (or the patient) have to pay any diagnostic/lab fee?

Yes (1) No (2) **→ *Go to C46***

Don’t remember (3) **→ *Go to C46*** Don’t know (4) **→ *Go to C46***

C45. How much did you pay for diagnostic/lab tests?

(*don’t know=979797)*

C46. During the second visit, did you (or the patient) pay any diagnostic/lab fee ?

Yes (1) No (2) **→ *Go to C48***

Don’t remember (3) **→ *Go to C48*** Don’t know (4) **→ *Go to C48***

C47. How much did you pay for diagnostic/lab tests?

(*don’t know=979797)*

C48. During the second visit, did you (or the patient) pay any fee for transportation (from your

house to the second facility and back to your house)?

Yes (1) No (2) **→ *Go to C50***

Don’t remember (3) **→ *Go to C50*** Don’t know (4) **→ *Go to C50***

C49. How much did you pay for the round trip (forth and back to your house)?

(*don’t know=979797)*

C50. During the second visit, did you (or the patient) pay any fee for hospitalization?

Yes (1) No (2) **→ *Go to C52***

Don’t remember (3) **→ *Go to C52*** Don’t know (4) **→ *Go to C52***

C51. How much did you pay for hospitalization?

(*don’t know=979797)*

C52. During the second visit, did you (or the patient) pay any other fee outside what we have

mentioned above?

Yes (1) No (2) **→ *Go to C55***

Don’t remember (3) **→ *Go to C55*** Don’t know (4) **→ *Go to C55***

C53. Please specify…………………………………………………………………………………..

C54. How much did it cost? (*don’t know=979797)*

C55. In case you (or the patient) did not remember one or more of the fees borne, how much money

did you spent **in total** during your second visit? (*don’t remember=979797)*

C56. As you (the patient) did not remember the total amount, was it between?

0-100 ZMW (1) 101-500 ZMW (2) 501-1,000 ZMW (3)

1,001-2,000 ZMW (4) over 2,000 ZMW (5)

C57. As to your (the patient) memory, what was the main means of transportation you (the patient) used to make the second visit?

On foot/walking (1) Bicycle (2) Motorcycle (3)

Minibus (4) Private car (5) Horse cart (6)

Boat (7) Other, specify(8) …………………………………

C58. How long did you (the patient) take to get to the place of the second visit (consider round trip)?

Minutes (*don’t know=979)*

C59. Excluding you (the patient), how many people in total accompanied you (the patient) to the place of the second visit?

Number 🡪 **if 00 or 97 Go to C63**

(*don’t know=97 ; 00 if no one accompanied the patient )*

C60. Out of the total in **C59.**, how many people used a separate means of transportation (*people that did not travelled using simultaneously the same means of transportation as you (the patient*)?

Number (*don’t know=97)*

C61. On average, how much did a round trip cost to each accompany person (*approximate please*)?

ZMK (*don’t know=979797 ; no expense=000000)*

C62. Excluding you (the patient), on average, how much did each accompany person spend on food/water

during your second visit (*approximate please*)?

ZMK (*don’t know=979797; no expense=000000)*

C63. Out of the total in **C59.**, how many accompany person booked lodging during your second visit? (*approximate please*)

Number (**don’t know=97 or if 00 🡪 Go to 65***)*

C64. how much did each accompany person spend on lodging during your second visit (*approximate*

*please*)?

ZMK (*don’t know=979797; no expense=000000)*

*I would like to ask questions regarding the* ***third visit*** *you have done in seeking care for your cholera*

*illness*

C65. As to your memory, where did you seek treatment?

District hospital (1) Health centre/post (2) Private facility (3)

Traditional healer (4) Cholera treatment centre (5)

Cholera treatment unit (6)

Other, specify (7) …………………………………………………………..

C66. During the third visit, did you (or the patient) have to buy food and/or water for yourself

(the patient)?

Yes (1) No (2) **→ *Go to C68***

Don’t remember (3) **→ *Go to C68*** Don’t know (4) **→ *Go to C68***

C67. How much did you pay for the food and/or water during your third visit?

(*don’t know =979797)*

C68. During the third visit, did you (or the patient) pay any consultation fee (to doctors,

nurses, healers)?

Yes (1) No (2) **→ *Go to C70***

Don’t remember (3) **→ *Go to C70*** Don’t know (4) **→ *Go to C70***

C69. How much did you pay for consultations? (*don’t know =979797)*

C70. During the third visit, did you (or the patient) pay any medicine/drugs?

Yes (1) No (2) **→ *Go to C72***

Don’t remember (3) **→ *Go to C72*** Don’t know (4) **→ *Go to C72***

C71. How much did you pay for medicine/drugs (excluding herbs and roots from healers)?

(*don’t know=979797)*

C72. During the third visit, did you (or the patient) pay any fee to traditional healers?

Yes (1) No (2) **→ *Go to C74***

Don’t remember (3) **→ *Go to C74*** Don’t know (4) **→ *Go to C74***

C73. How much did you pay for roots and herbs from healers (excluding modern medicine/drugs)?

(*don’t know=979797)*

C74. During the third visit, did you (or the patient) have to pay any diagnostic/lab fee?

Yes (1) No (2) **→ *Go to C76***

Don’t remember (3) **→ *Go to C76*** Don’t know (4) **→ *Go to C76***

C75. How much did you pay for diagnostic/lab tests?

(*don’t know=979797)*

C76. During the third visit, did you (or the patient) pay any diagnostic/lab fee ?

Yes (1) No (2) **→ *Go to C78***

Don’t remember (3) **→ *Go to C78*** Don’t know (4) **→ *Go to C78***

C77. How much did you pay for diagnostic/lab tests?

(*don’t know=979797)*

C78. During the second visit, did you (or the patient) have to pay any fee for transportation (from your

house to the third facility and back to your house)?

Yes (1) No (2) **→ *Go to C80***

Don’t remember (3) **→ *Go to C80*** Don’t know (4) **→ *Go to C80***

C79. How much did you pay for the round trip (forth and back to your house)?

(*don’t know=979797)*

C80. During the third visit, did you (or the patient) pay any fee for hospitalization?

Yes (1) No (2) **→ *Go to C82***

Don’t remember (3) **→ *Go to C82*** Don’t know (4) **→ *Go to C82***

C81. How much did you pay for hospitalization?

(*don’t know=979797)*

C82. During the third visit, did you (or the patient) have to pay any other fee outside what we have

mentioned above ?

Yes (1) No (2) **→ *Go to C85***

Don’t remember (3) **→ *Go to C85*** Don’t know (4) **→ *Go to C85***

C83. Please specify…………………………………………………………………………………..

C84. How much did it cost? (*don’t know=979797)*

C85. In case you (or the patient) did not remember one or more of the fees borne, how much money

did you spent **in total** during your third visit? (*don’t remember=989898)*

C86. As you (the patient) did not remember the total amount, was it between?

0-100 ZMW (1) 101-500 ZMW (2) 501-1,000 ZMW (3)

1,001-2,000 ZMW (4) over 2,000 ZMW (5)

C87. As to your (the patient) memory, what was the main means of transportation you (the patient)

during his third visit?

On foot/walking (1) Bicycle (2) Motorcycle (3)

Minibus (4) Private car (5) Horse cart (6)

Boat (7) Other, specify (8) …………………………………

C88. How long did you (the patient) take to get to the place of your (the patient) third visit (consider round trip)?

Minutes (*don’t know=979)*

**C89**. Excluding you (the patient), how many people in total accompanied you (the patient) to the place of the third visit?

Number 🡪 **if 00 or 97 Go to C93**

(*don’t know=97 ; 00 if no one accompanied the patient )*

C90. Out of the total in **C89.**, how many people used a separate means of transportation (*people that did not travelled using simultaneously the same means of transportation as you (the patient*)?

Number (*don’t know=97)*

C91. On average, how much did a round trip cost to each accompany person (*approximate please*)?

ZMK (*don’t know=979797 ; no expense=000000)*

C92. Excluding you (the patient), on average, how much did each accompany person spend on food/water

during your third visit (*approximate please*)?

ZMK (*don’t know=979797; no expense=000000)*

C93. Out of the total in **C89.**, how many accompany person booked lodging during your third visit? (*please approximate*) Number (**don’t know=97 or if 00 🡪 Go to 95***)*

C94. How much did each accompany person spend on lodging during your third visit (*approximate*

*please*)? ZMK (*don’t know=979797; no expense=000000)*

*I would like to ask questions regarding the* ***fourth visit*** *you have done in seeking care for your cholera*

*illness*

C95. As to your memory, where did you seek treatment?

District hospital (1) Health centre/post (2) Private facility (3)

Traditional healer (4) Cholera treatment centre (5)

Cholera treatment unit (6) Other, specify (7) …………………………………………

C96. During the fourth visit, did you (or the patient) buy food and/or water for yourself

(the patient)?

Yes (1) No (2) **→ *Go to C98***

Don’t remember (3) **→ *Go to C98*** Don’t know (4) **→ *Go to C98***

C97. How much did you pay for the food and/or water during your fourth visit?

(*don’t know =979797)*

C98. During the fourth visit, did you (or the patient) pay any consultation fee (to doctors,

nurses, healers)?

Yes (1) No (2) **→ *Go to C100***

Don’t remember (3) **→ *Go to C100*** Don’t know (4) **→ *Go to C100***

C99. How much did you pay for consultations? (*don’t know =979797)*

C100. During the fourth visit, did you (or the patient) pay any medicine/drugs ?

Yes (1) No (2) **→ *Go to C102***

Don’t remember (3) **→ *Go to C102*** Don’t know (4) **→ *Go to C102***

C101. How much did you pay for medicine/drugs (excluding herbs and roots from healers)?

(*don’t know=979797)*

C102. During the fourth visit, did you (or the patient) pay any fee to traditional healers?

Yes (1) No (2) **→ *Go to C104***

Don’t remember (3) **→ *Go to C104*** Don’t know (4) **→ *Go to C104***

C103. How much did you pay for roots and herbs from healers (excluding modern medicine/drugs)?

(*don’t know=979797)*

C104. During the fourth visit, did you (or the patient) pay any diagnostic/lab fee ?

Yes (1) No (2) **→ *Go to C106***

Don’t remember (3) **→ *Go to C106*** Don’t know (4) **→ *Go to C106***

C105. How much did you pay for diagnostic/lab tests?

(*don’t know=979797)*

C106. During the fourth visit, did you (or the patient) pay any diagnostic/lab fee ?

Yes (1) No (2) **→ *Go to C108***

Don’t remember (3) **→ *Go to C108*** Don’t know (4) **→ *Go to C108***

C107. How much did you pay for diagnostic/lab tests?

(*don’t know=979797)*

C108. During the fourth visit, did you (or the patient) pay any fee for transportation (from your

house to the third facility and back to your house)?

Yes (1) No (2) **→ *Go to C110***

Don’t remember (3) **→ *Go to C110*** Don’t know (4) **→ *Go to C110***

C109. How much did you pay for the round trip (forth and back to your house)?

(*don’t know=979797)*

C110. During the fourth visit, did you (or the patient) have to pay any fee for hospitalization?

Yes (1) No (2) **→ *Go to C112***

Don’t remember (3) **→ *Go to C112*** Don’t know (4) **→ *Go to C112***

C111. How much did you pay for hospitalization?

(*don’t know=979797)*

C112. During the fourth visit, did you (or the patient) have to pay any other fee outside what we have

mentioned above?

Yes (1) No (2) **→ *Go to C115***

Don’t remember (3) **→ *Go to C115*** Don’t know (4) **→ *Go to C115***

C113. Please specify…………………………………………………………………………………..

C114. How much did it costs? (*don’t know=979797)*

C115. In case you (or the patient) did not remember one or more of the fees borne, how much money

did you spent **in total** during your fourth visit? (*don’t remember=979797)*

C116. As you (the patient) did not remember the total amount, was it between?

0-100 ZMW (1) 101-500 ZMW (2) 501-1,000 ZMW (3)

1,001-2,000 ZMW (4) over 2,000 ZMW (5)

C117. As to your (the patient) memory, what was the main means of transportation you (the patient) used to make the fourth visit?

On foot/walking (1) Bicycle (2) Motorcycle (3)

Minibus (4) Private car (5) Horse cart (6)

Boat (7) Other, specify (8) …………………………………

C118. How long did you (the patient) take to get to the place of your (the patient) fourth visit (consider round trip)?

Minutes (*don’t know=979)*

**C119**. Excluding you (the patient), how many people in total accompanied you (the patient) to the place of the fourth visit? Number 🡪 **if 00 or 97 Go to C123**

(*don’t know=97 ; 00 if no one accompanied the patient )*

C120. Out of the total in **C119.**, how many people used a separate means of transportation (*people that did not travelled using simultaneously the same means of transportation as you (the patient*)? Number (*don’t know=97)*

C121. On average, how much did a round trip cost to each accompany person (*approximate please*)?

ZMK (*don’t know=979797 ; no expense=000000)*

C122. Excluding you (the patient), on average, how much did each accompany person spend on food/water

during your fourth visit (*approximate please*)?

ZMK (*don’t know=979797; no expense=000000)*

C123. Out of the total in **C119.**, how many accompany person booked lodging during your fourth visit? (*approximate please*) Number (**don’t know=97 or if 00 🡪 Go to 125***)*

C124. How much did each accompany person spend on lodging during your fourth visit (*approximate*

*please*)? ZMK (*don’t know=979797; no expense=000000)*

*I would like to ask questions regarding the* ***fifth visit*** *you have done in seeking care for your cholera*

*illness*

C125. As to your memory, where did you seek treatment?

District hospital (1) Health centre/post (2) Private facility (3)

Traditional healer (4) Cholera treatment centre (5)

Cholera treatment unit (6) Other, specify (7) …………………………………………

C126. During the fifth visit, did you (or the patient) buy food and/or water for yourself (the patient)?

Yes (1) No (2) **→ *Go to C128***

Don’t remember (3) **→ *Go to C128*** Don’t know (4) **→ *Go to C128***

C127. How much did you pay for the food and/or water during your fourth visit?

(*don’t know =979797)*

C128. During the fifth visit, did you (or the patient) pay any consultation fee (to doctors, nurses, healers)?

Yes (1) No (2) **→ *Go to C130***

Don’t remember (3) **→ *Go to C130*** Don’t know (4) **→ *Go to C130***

C129. How much did you pay for consultations? (*don’t know =979797)*

C130. During the fifth visit, did you (or the patient) pay any medicine/drugs?

Yes (1) No (2) **→ *Go to C132***

Don’t remember (3) **→ *Go to C132*** Don’t know (4) **→ *Go to C132***

C131. How much did you pay for medicine/drugs (excluding herbs and roots from healers)?

(*don’t know=979797)*

C132. During the fifth visit, did you (or the patient) pay any fee to traditional healers?

Yes (1) No (2) **→ *Go to C134***

Don’t remember (3) **→ *Go to C134*** Don’t know (4) **→ *Go to C134***

C133. How much did you pay for roots and herbs from healers (excluding modern medicine/drugs)?

(*don’t know=979797)*

C134. During the fifth visit, did you (or the patient) pay any diagnostic/lab fee ?

Yes (1) No (2) **→ *Go to C136***

Don’t remember (3) **→ *Go to C136*** Don’t know (4) **→ *Go to C136***

C135. How much did you pay for diagnostic/lab tests?

(*don’t know=979797)*

C136. During the fifth visit, did you (or the patient) pay any diagnostic/lab fee ?

Yes (1) No (2) **→ *Go to C138***

Don’t remember (3) **→ *Go to C138*** Don’t know (4) **→ *Go to C138***

C137. How much did you pay for diagnostic/lab tests?

(*don’t know=979797)*

C138. During the fifth visit, did you (or the patient) pay any fee for transportation (from your

house to the third facility and back to your house)?

Yes (1) No (2) **→ *Go to C140***

Don’t remember (3) **→ *Go to C140*** Don’t know (4) **→ *Go to C140***

C139. How much did you pay for the round trip (forth and back to your house)?

(*don’t know=979797)*

C140. During the fifth visit, did you (or the patient) pay any fee for hospitalization?

Yes (1) No (2) **→ *Go to C142***

Don’t remember (3) **→ *Go to C142*** Don’t know (4) **→ *Go to C142***

C141. How much did you pay for hospitalization?

(*don’t know=979797)*

C142. During the fifth visit, did you (or the patient) pay any other fee outside what we have

mentioned above ?

Yes (1) No (2) **→ *Go to C145***

Don’t remember (3) **→ *Go to C145*** Don’t know (4) **→ *Go to C145***

C143. Please specify…………………………………………………………………………………..

C144. How much did it costs? (*don’t know=979797)*

C145. In case you (or the patient) did not remember one or more of the fees borne, how much money

did you spent **in total** during your fifth visit? (*don’t remember=979797)*

C146. As you (the patient) did not remember the total amount, was it between?

0-100 ZMW (1) 101-500 ZMW (2) 501-1,000 ZMW (3)

1,001-2,000 ZMW (4) over 2,000 ZMW (5)

C147. As to your (the patient) memory, what was the main means of transportation you (the patient) used to make the fifth visit?

On foot/walking (1) Bicycle (2) Motorcycle (3)

Minibus (4) Private car (5) Horse cart (6)

Boat (7) Other, specify (8) …………………………………

C148. How long did you (the patient) take to get to the place of your (the patient) fifth visit (consider round trip) Minutes (*don’t know=979)*

**C149**. Excluding you (the patient), how many people in total accompanied you (the patient) to the place of the fifth visit? Number 🡪 **if 00 or 97 Go to C153**

(*don’t know=97 ; 00 if no one accompanied the patient )*

C150. Out of the total in **C149.**, how many people used a separate means of transportation (*people that did not travelled using simultaneously the same means of transportation as you (the patient*)? Number (*don’t know=97)*

C151. On average, how much did a round trip cost to each accompany person (*approximate please*)?

ZMK (*don’t know=979797 ; no expense=000000)*

C152. Excluding you (the patient), on average, how much did each accompany person spend on food/water

during your fifth visit (*approximate please*)?

ZMK (*don’t know=979797; no expense=000000)*

C153. Out of the total in **C149.**, how many accompany person booked lodging during your fifth visit? (*approximate please*) Number (**don’t know=97 or if 00 🡪 Go to C155***)*

C154. How much did each accompany person spend on lodging during your fifth visit (*approximate*

*please*)? ZMK (*don’t know=979797; no expense=000000)*

**Section E: Indirect costs: Patient productivity losses & opportunity costs**

*Now, I would like to ask you about the ways you (or the patient’s) cholera illness affected those*

*around you. Sometimes, people with cholera feel so sick that they cannot perform any of their usual*

*activities. Instead, they have to rest and stay in bed. Other people around the patient may not be as*

*sick, and can still do their usual activities. But they may not be able to perform their activities as*

*well as normal because they may have to help or take care of the patient*.

*When you answer these questions, please think about the entire time period when you were sick with*

*cholera, Starting before you (or the patient) went to the health facility for diagnosis*.

E1. In total, how many days have you (or the patient) been sick with cholera since the symptoms started? Days (*Don’t know=97 ; Not applicable=99*)

E2. How many days were you (or the patient) **completely unable** to do any of your usual activities while sick? Days (*Don’t know=97 ; Not applicable=99*)

E3. How many days were you (or the patient) able to perform **some but not all** of your usual activities while sick? Days (*Don’t know=97 ; Not applicable=99*)

E4. How many days were you (or the patient) able to perform **all** of your usual activities while sick? Days (*Don’t know=97 ; Not applicable=99*)

*Interviewers, please verify that total number of days (E2+E3+E4) equal E1; if not, please go back to E1 and make all necessary revisions*

E5. What would you (or the patient) be mainly doing if you had not been sick?

Working for an employer, informal (1) Working for self (2)

Government worker (3) Private worker, formal (4)

Housework (cooking, cleaning, etc) (5) Going to school (6)

Leisure or play time (7) Private worker, informal (8)

Other, specify (9) …………………………………………

E6. Do you (or the patient) work for a wage (cash or in kind)?

Yes, paid in cash (1) 🡪 **Go to E7** Yes, paid in-kind (2) 🡪 **Go to E8**

No (3) 🡪**Go to E11**

E7. How much was you paid a day? ZMK

(*Don’t know=979797 ; Refusal=999999*)

E8. Please specify the approximate value per day? ZMK

(*Don’t know=979797 ; Refusal=999999*)

E9. If you work for a wage, did your (or the patient’s) employer or boss pay for the days you were sick but did not come to work

Yes (1) No (2) **Go to E12**  Don’t know (9) 🡪 **Go to E12**

E10. How many sick days did your (or the patient’s) employer or boss pay for you?

Days (*Don’t know=97 ; Not applicable=99*)

E11. In total, what is your (or the patient’s) monthly income ZMK

(*Don’t know=979797 ; Refusal=999999*)

E12. In total, how many days of income did you (or the patient’s) loose because of this illness since it started?

Days (*Don’t know=97 ; Patient did not return to work=98; Not applicable=99*)

**Section F: Indirect costs: Caretakers & Substitute labor costs**

*Now I would like to understand if anyone helped you (or the patient) while you were sick. This includes 2 types of people:*

1. *Someone who may have been paid to complete your usual tasks for you*
2. *Someone who had to help take care of you (or the patient) while you were sick*

F1. Were you (or the patient) so sick with cholera that someone had to help you (or the patient)

in any way?

Yes (1) No (2) **Go to G1** Don’t know (9) 🡪 **Go to G1**

F2. In total, how many people cared for you (or the patient) or did your usual activities for you while you were sick with cholera?

people (*Don’t know=97*)

*Questions* ***F3 to F14*** *record productivity losses for the* ***first helper/caretaker***

F3. What is your (the patient) relationship with the person who helped you (the patient) while sick?

Mother (1) Father (2) Husband/partner(3) Wife/partner (4) Brother (5) Sister (6)

Hired help (7) Grandmother/father (8) Neighbor (9)

Other, specify (10) …………………………………………

F4. Is this helper a member of your household?

Yes (1) No (2) Don’t know (9)

F5. How old is this helper?

years (*Don’t know=97* )

F6. How many days or hours did he/she helped you (or the patient)?

Days (*Don’t know=97*) or Hours (*Don’t know=9797*)

F7. Was this helper paid to help you (or the patient)?

Yes (1) No (2)**🡪 Go to F9** Don’t know (9) **🡪 Go to F9**

F8. How much was this helper **paid per day** to help the patient?

ZMK (*Don’t know=979797 ; refusal=999999*)

F9. Did this helper perform?

All of the patient’s activities (1) Some of the patient’s activities (2)

None of the patient’s activities (3) Do not know (9)

F10. Did the helper cut back on his/her own usual activities?

Yes (1) No (2) **🡪 Go to** **F13** Do not know (9) **🡪 Go to** **F13**

F11. How many days or hours did the helper cut back on his/her own activities?

Days (*Don’t know=97*) or Hours (*Don’t know=9797*)

F12. Was the helper able to do some or none of his/her own usual activities?

Able to do some of his/her usual activities (1)

None of of his/her usual activities (2)

Do not know (9)

F13. What would the helper have been doing mainly if s/he had not been caring for the sick patient?

Working for an employer, informal (1) Working for self (2)

Government worker (3) Private worker, formal (4)

Housework (cooking, cleaning…) (5) Going to school (6)

Leisure or play time (7) Private worker, informal (8)

Other, specify (9) …………………………………………

F14. If helper works for a wage, how much is s/he normally paid per day for his/her?

ZMK (*Don’t know=979797 ; refusal=999999*)

*Questions* ***F15 to F26*** *record productivity losses for the* ***second helper/caretaker***

F15. What is your (the patient) relationship with the person who helped you (the patient) while sick?

Mother (1) Father (2) Husband/partner(3) Wife/partner (4) Brother (5) Sister (6)

Hired help (7) Grandmother/father (8) Neighbor (9)

Other, specify (10) …………………………………………

F16. Is this helper a member of your household?

Yes (1) No (2) Don’t know (9)

F17. How old is this helper?

years (*Don’t know=97* )

F18. How many days or hours did he/she helped you (or the patient)?

Days (*Don’t know=97*) or Hours (*Don’t know=9797*)

F19. Was this helper paid to help you (or the patient)?

Yes (1) No (2)**🡪 Go to F21** Don’t know (9) **🡪 Go to F21**

F20. How much was this helper **paid per day** to help the patient?

ZMK (*Don’t know=979797 ; refusal=999999*)

F21. Did this helper perform?

All of the patient’s activities (1) Some of the patient’s activities (2)

None of the patient’s activities (3) Do not know (9)

F22. Did the helper cut back on his/her own usual activities?

Yes (1) No (2) **🡪 Go to** **F25** Do not know (9) **🡪 Go to** **F25**

F23. How many days or hours did the helper cut back on his/her own activities?

Days (*Don’t know=97*) or Hours (*Don’t know=9797*)

F24. Was the helper able to do some or none of his/her own usual activities?

Able to do some of his/her usual activities (1)

None of of his/her usual activities (2)

Do not know (9)

F25. What would the helper have been doing mainly if s/he had not been caring for the sick patient?

Working for an employer, informal (1) Working for self (2)

Government worker (3) Private worker, formal (4)

Housework (cooking, cleaning…) (5) Going to school (6)

Leisure or play time (7) Private worker, informal (8)

Other, specify (9) …………………………………………

F26. If helper works for a wage, how much is s/he normally paid per day for his/her?

ZMK (*Don’t know=979797 ; refusal=999999*)

*Questions* ***F27 to F38*** *record productivity losses for the* ***third helper/caretaker***

F27. What is your (the patient) relationship with the person who helped you (the patient) while sick?

Mother (1) Father (2) Husband/partner (3)

Wife/partner (4) Brother (5) Sister (6)

Hired help (7) Grandmother/father (8) Neighbor (9)

Other, specify (10) …………………………………………

F28. Is this helper a member of your household?

Yes (1) No (2) Don’t know (9)

F29. How old is this helper?

years (*Don’t know=97)*

F30. How many days or hours did he/she helped you (or the patient)?

Days (*Don’t know=97*) or Hours (*Don’t know=9797*)

F31. Was this helper paid to help you (or the patient)?

Yes (1) No (2)**🡪 Go to F33** Don’t know (9) **🡪 Go to F33**

F32. How much was this helper **paid per day** to help the patient?

ZMK (*Don’t know=979797 ; refusal=999999*)

F33. Did this helper perform?

All of the patient’s activities (1) Some of the patient’s activities (2)

None of the patient’s activities (3) Do not know (9)

F34. Did the helper cut back on his/her own usual activities?

Yes (1) No (2) **🡪 Go to** **F37** Do not know (9) **🡪 Go to** **F37**

F35. How many days or hours did the helper cut back on his/her own activities?

Days (*Don’t know=97*) or Hours (*Don’t know=9797*)

F36. Was the helper able to do some or none of his/her own usual activities?

Able to do some of his/her usual activities (1)

None of of his/her usual activities (2)

Do not know (9)

F37. What would the helper have been doing mainly if s/he had not been caring for the sick patient?

Working for an employer, informal (1) Working for self (2)

Government worker (3) Private worker, formal (4)

Housework (cooking, cleaning…) (5) Going to school (6)

Leisure or play time (7) Private worker, informal (8)

Other, specify (9) …………………………………………

F38. If helper works for a wage, how much is s/he normally paid per day for his/her?

ZMK (*Don’t know=979797 ; refusal=999999*)

*Questions* ***F39 to F50*** *record productivity losses for the* ***fourth helper/caretaker***

F39. What is your (the patient) relationship with the person who helped you (the patient) while sick?

Mother (1) Father (2) Husband/partner (3)

Wife/partner (4) Brother (5) Sister (6)

Hired help (7) Grandmother/father (8) Neighbor (9)

Other, specify (10) …………………………………………

F40. Is this helper a member of your household?

Yes (1) No (2) Don’t know (9)

F41. How old is this helper?

years (*Don’t know=97* )

F42. How many days or hours did he/she helped you (or the patient)?

Days (*Don’t know=97*) or Hours (*Don’t know=9797*)

F43. Was this helper paid to help you (or the patient)?

Yes (1) No (2)**🡪 Go to F45** Don’t know (9) **🡪 Go to F45**

F44. How much was this helper **paid per day** to help the patient?

ZMK (*Don’t know=979797 ; refusal=999999*)

F45. Did this helper perform?

All of the patient’s activities (1) Some of the patient’s activities (2)

None of the patient’s activities (3) Do not know (9)

F46. Did the helper cut back on his/her own usual activities?

Yes (1) No (2) **🡪 Go to** **F49** Do not know (9) **🡪 Go to** **F49**

F47. How many days or hours did the helper cut back on his/her own activities?

Days (*Don’t know=97*) or Hours (*Don’t know=9797*)

F48. Was the helper able to do some or none of his/her own usual activities?

Able to do some of his/her usual activities (1)

None of of his/her usual activities (2) Do not know (9)

F49. What would the helper have been doing mainly if s/he had not been caring for the sick patient?

Working for an employer, informal (1) Working for self (2)

Government worker (3) Private worker, formal (4)

Housework (cooking, cleaning…) (5) Going to school (6)

Leisure or play time (7) Private worker, informal (8)

Other, specify (9) …………………………………………

F50. If helper works for a wage, how much is s/he normally paid per day for his/her?

ZMK (*Don’t know=979797 ; refusal=999999*)

*Questions* ***F51 to F62*** *record productivity losses for the* ***fifth helper/caretaker***

F51. What is your (the patient) relationship with the person who helped you (the patient) while sick?

Mother (1) Father (2) Husband/partner (3)

Wife/partner (4) Brother (5) Sister (6)

Hired help (7) Grandmother/father (8) Neighbor (9)

Other, specify (10) …………………………………………

F52. Is this helper a member of your household?

Yes (1) No (2) Don’t know (9)

F53. How old is this helper?

years (*Don’t know=97* )

F54. How many days or hours did he/she helped you (or the patient)?

Days (*Don’t know=97*) or Hours (*Don’t know=9797*)

F55. Was this helper paid to help you (or the patient)?

Yes (1) No (2)**🡪 Go to F57** Don’t know (9) **🡪 Go to F57**

F56. How much was this helper **paid per day** to help the patient?

ZMK (*Don’t know=979797 ; refusal=999999*)

F57. Did this helper perform?

All of the patient’s activities (1) Some of the patient’s activities (2)

None of the patient’s activities (3) Do not know (9)

F58. Did the helper cut back on his/her own usual activities?

Yes (1) No (2) **🡪 Go to** **F61** Do not know (9) **🡪 Go to** **F61**

F59. How many days or hours did the helper cut back on his/her own activities?

Days (*Don’t know=97*) or Hours (*Don’t know=9797*)

F60. Was the helper able to do some or none of his/her own usual activities?

Able to do some of his/her usual activities (1)

None of of his/her usual activities (2)

Do not know (9)

F61. What would the helper have been doing mainly if s/he had not been caring for the sick patient?

Working for an employer, informal (1) Working for self (2)

Government worker (3) Private worker, formal (4)

Housework (cooking, cleaning, etc) (5) Going to school (6)

Leisure or play time (7) Private worker, informal (8)

Other, specify (9) …………………………………………

F62. If helper works for a wage, how much is s/he normally paid per day for his/her?

ZMK (*Don’t know=979797 ; refusal=999999*)

*Questions* ***F63 to F74*** *record productivity losses for the* ***sixth helper/caretaker***

F63. What is your (the patient) relationship with the person who helped you (the patient) while sick?

Mother (1) Father (2) Husband/partner (3)

Wife/partner (4) Brother (5) Sister (6)

Hired help (7) Grandmother/father (8) Neighbor (9)

Other, specify (10) …………………………………………

F64. Is this helper a member of your household?

Yes (1) No (2) Don’t know (9)

F65. How old is this helper?

years (*Don’t know=97* )

F66. How many days or hours did he/she helped you (or the patient)?

Days (*Don’t know=97*) or Hours (*Don’t know=9797*)

F67. Was this helper paid to help you (or the patient)?

Yes (1) No (2)**🡪 Go to F69** Don’t know (9) **🡪 Go to F69**

F68. How much was this helper **paid per day** to help the patient?

ZMK (*Don’t know=979797 ; refusal=999999*)

F69. Did this helper perform?

All of the patient’s activities (1) Some of the patient’s activities (2)

None of the patient’s activities (3) Do not know (9)

F70. Did the helper cut back on his/her own usual activities?

Yes (1) No (2) **🡪 Go to** **F73** Do not know (9) **🡪 Go to** **F73**

F71. How many days or hours did the helper cut back on his/her own activities?

Days (*Don’t know=97*) or Hours (*Don’t know=9797*)

F72. Was the helper able to do some or none of his/her own usual activities?

Able to do some of his/her usual activities (1)

None of of his/her usual activities (2)

Do not know (9)

F73. What would the helper have been doing mainly if s/he had not been caring for the sick patient?

Working for an employer, informal (1) Working for self (2)

Government worker (3) Private worker, formal (4)

Housework (cooking, cleaning, etc) (5) Going to school (6)

Leisure or play time (7) Private worker, informal (8)

Other, specify (9) …………………………………………

F74. If helper works for a wage, how much is s/he normally paid per day for his/her?

ZMK (*Don’t know=979797 ; refusal=999999*)

*Questions* ***F75 to F86*** *record productivity losses for the* ***seventh helper/caretaker***

F75. What is your (the patient) relationship with the person who helped you (the patient) while sick?

Mother (1) Father (2) Husband/partner (3)

Wife/partner (4) Brother (5) Sister (6)

Hired help (7) Grandmother/father (8) Neighbor (9)

Other, specify (10) …………………………………………

F76. Is this helper a member of your household?

Yes (1) No (2) Don’t know (9)

F77. How old is this helper?

years (*Don’t know=97* )

F78. How many days or hours did he/she helped you (or the patient)?

Days (*Don’t know=97*) or Hours (*Don’t know=9797*)

F79. Was this helper paid to help you (or the patient)?

Yes (1) No (2)**🡪 Go to F81** Don’t know (9) **🡪 Go to F81**

F80. How much was this helper **paid per day** to help the patient?

ZMK (*Don’t know=979797 ; refusal=999999*)

F81. Did this helper perform?

All of the patient’s activities (1) Some of the patient’s activities (2)

None of the patient’s activities (3) Do not know (9)

F82. Did the helper cut back on his/her own usual activities?

Yes (1) No (2) **🡪 Go to** **F85** Do not know (9) **🡪 Go to** **F85**

F83. How many days or hours did the helper cut back on his/her own activities?

Days (*Don’t know=97*) or Hours (*Don’t know=9797*)

F84. Was the helper able to do some or none of his/her own usual activities?

Able to do some of his/her usual activities (1)

None of of his/her usual activities (2)

Do not know (9)

F85. What would the helper have been doing mainly if s/he had not been caring for the sick patient?

Working for an employer, informal (1) Working for self (2)

Government worker (3) Private worker, formal (4)

Housework (cooking, cleaning, etc) (5) Going to school (6)

Leisure or play time (7) Private worker, informal (8)

Other, specify (9) …………………………………………

F86. If helper works for a wage, how much is s/he normally paid per day for his/her?

ZMK (*Don’t know=979797 ; refusal=999999*)

*Questions* ***F86 to F97*** *record productivity losses for the* ***eighth helper/caretaker***

F86. What is your (the patient) relationship with the person who helped you (the patient) while sick?

Mother (1) Father (2) Husband/partner (3)

Wife/partner (4) Brother (5) Sister (6)

Hired help (7) Grandmother/father (8) Neighbor (9)

Other, specify (10) …………………………………………

F87. Is this helper a member of your household?

Yes (1) No (2) Don’t know (9)

F88. How old is this helper?

years (*Don’t know=97* )

F89. How many days or hours did he/she helped you (or the patient)?

Days (*Don’t know=97*) or Hours (*Don’t know=9797*)

F90. Was this helper paid to help you (or the patient)?

Yes (1) No (2)**🡪 Go to F92** Don’t know (9) **🡪 Go to F92**

F91. How much was this helper **paid per day** to help the patient?

ZMK (*Don’t know=979797 ; refusal=999999*)

F92. Did this helper perform?

All of the patient’s activities (1) Some of the patient’s activities (2)

None of the patient’s activities (3) Do not know (9)

F93. Did the helper cut back on his/her own usual activities?

Yes (1) No (2) **🡪 Go to** **F96** Do not know (9) **🡪 Go to** **F96**

F94. How many days or hours did the helper cut back on his/her own activities?

Days (*Don’t know=97*) or Hours (*Don’t know=9797*)

F95. Was the helper able to do some or none of his/her own usual activities?

Able to do some of his/her usual activities (1)

None of of his/her usual activities (2)

Do not know (9)

F96. What would the helper have been doing mainly if s/he had not been caring for the sick patient?

Working for an employer, informal (1) Working for self (2)

Government worker (3) Private worker, formal (4)

Housework (cooking, cleaning, etc) (5) Going to school (6)

Leisure or play time (7) Private worker, informal (8)

Other, specify (9) …………………………………………

F97. If helper works for a wage, how much is s/he normally paid per day for his/her?

ZMK (*Don’t know=979797 ; refusal=999999*)

*Questions* ***F98 to F109*** *record productivity losses for the* ***ninth helper/caretaker***

F98. What is your (the patient) relationship with the person who helped you (the patient) while sick?

Mother (1) Father (2) Husband/partner (3)

Wife/partner (4) Brother (5) Sister (6)

Hired help (7) Grandmother/father (8) Neighbor (9)

Other, specify (10) …………………………………………

F99. Is this helper a member of your household?

Yes (1) No (2) Don’t know (9)

F100. How old is this helper?

years (*Don’t know=97* )

F101. How many days or hours did he/she helped you (or the patient)?

Days (*Don’t know=97*) or Hours (*Don’t know=9797*)

F102. Was this helper paid to help you (or the patient)?

Yes (1) No (2)**🡪 Go to F104** Don’t know (9) **🡪 Go to F104**

F103. How much was this helper **paid per day** to help the patient?

ZMK (*Don’t know=979797 ; refusal=999999*)

F104. Did this helper perform?

All of the patient’s activities (1) Some of the patient’s activities (2)

None of the patient’s activities (3) Do not know (9)

F105. Did the helper cut back on his/her own usual activities?

Yes (1) No (2) **🡪 Go to** **F108** Do not know (9) **🡪 Go to** **F108**

F106. How many days or hours did the helper cut back on his/her own activities?

Days (*Don’t know=97*) or Hours (*Don’t know=9797*)

F107. Was the helper able to do some or none of his/her own usual activities?

Able to do some of his/her usual activities (1)

None of of his/her usual activities (2)

Do not know (9)

F108. What would the helper have been doing mainly if s/he had not been caring for the sick patient?

Working for an employer, informal (1) Working for self (2)

Government worker (3) Private worker, formal (4)

Housework (cooking, cleaning, etc) (5) Going to school (6)

Leisure or play time (7) Private worker, informal (8)

Other, specify (9) …………………………………………

F109. If helper works for a wage, how much is s/he normally paid per day for his/her?

ZMK (*Don’t know=979797 ; refusal=999999*)

*Questions* ***F110 to F121*** *record productivity losses for the* ***tenth helper/caretaker***

F110. What is your (the patient) relationship with the person who helped you (the patient) while sick?

Mother (1) Father (2) Husband/partner (3)

Wife/partner (4) Brother (5) Sister (6)

Hired help (7) Grandmother/father (8) Neighbor (9)

Other, specify (10) …………………………………………

F111. Is this helper a member of your household?

Yes (1) No (2) Don’t know (9)

F112. How old is this helper?

years (*Don’t know=97* )

F113. How many days or hours did he/she helped you (or the patient)?

Days (*Don’t know=97*) or Hours (*Don’t know=9797*)

F114. Was this helper paid to help you (or the patient)?

Yes (1) No (2)**🡪 Go to F116** Don’t know (9) **🡪 Go to F116**

F115. How much was this helper **paid per day** to help the patient?

ZMK (*Don’t know=979797 ; refusal=999999*)

F116. Did this helper perform?

All of the patient’s activities (1) Some of the patient’s activities (2)

None of the patient’s activities (3) Do not know (9)

F1117. Did the helper cut back on his/her own usual activities?

Yes (1) No (2) **🡪 Go to** **F120** Do not know (9) **🡪 Go to** **F120**

F118. How many days or hours did the helper cut back on his/her own activities?

Days (*Don’t know=97*) or Hours (*Don’t know=9797*)

F119. Was the helper able to do some or none of his/her own usual activities?

Able to do some of his/her usual activities (1)

None of of his/her usual activities (2)

Do not know (9)

F120. What would the helper have been doing mainly if s/he had not been caring for the sick patient?

Working for an employer, informal (1) Working for self (2)

Government worker (3) Private worker, formal (4)

Housework (cooking, cleaning, etc) (5) Going to school (6)

Leisure or play time (7) Private worker, informal (8)

Other, specify (9) …………………………………………

F121. If helper works for a wage, how much is s/he normally paid per day for his/her?

ZMK (*Don’t know=979797 ; refusal=999999*)

**Section G: Financial impact of cholera and household socioeconomic status**

*There a few more questions I would like to ask you to understand how this cholera event impacted your (or the patient’s) financial situation*

G1. Did you (or the patient) or your household have to adopt some coping strategies in order to

pay for your (or the patient) healthcare because of cholera illness ?

Yes (1) No (2)**🡪 Go to G3** Don’t know (9)**🡪 Go to G3**

G1. Which strategy(ies) did you (or the patient) or the household adopted (*multiple choices allowed*)?

Sold land Yes (1) No (2)

Sold livestock Yes (1) No (2)

Sold agricultural products Yes (1) No (2)

Sold household assets (*bicycle, TV, clothe,s radio…)* Yes (1) No (2)

Sold jewelry Yes (1) No (2)

Sold in advance future harvest(*below its market value*) Yes (1) No (2)

Borrow money (*to household members and friends*) Yes (1) No (2)

Use of savings till exhaustion Yes (1) No (2)

Reducing household expenses Yes (1) No (2)

G3. Compared to before you (the patient) visited the health facility, how is your (the patient) physical wellness

today?

Worse (1) The same (2) Better (3)

G4. Do you feel that you (or the patient) has fully recovered from cholera ?

Yes (1) No (2)**🡪 Go to H**  Not sure (3)**🡪 Go to H**

G5. What is the main source of drinking water in your household?

River/barrage (1) Pond/lake (2) Borhole (3)

Natural spring (4) Rain water (5) Bottle water (6)

Piped water in the household (7) Public piped water (8)

Traditional borehole in the household (9) Public traditional borehole (10)

Other, specify (11) ………………………………………………

G6. What is the material of the roof of the main house of the household?

Straw (1) Soil (2)

Metal sheet (3) Cement/concrete (4)

Tiles (5) Other, specify (6) ……………………………

G7. What type of toilets do members of your household usually use?

Individual flushing toilets (1) Common flushing toilets (2) Latrines (3)

Ventilated latrines (4) No toilets/nature (5)

Other (6) Specify……………………………………………………..

G8. In which material are the walls of the main house of the household made?

Soil (1) Cement/concrete (2) Metal sheets (3)

Straw/wood (4) Wild stone (5)

Other, specify (6) ……………………………………………………..

G9. Do you have any of the following assets (in working condition) in your household?

Electricity Yes (1) No (0)

Radio Yes (1) No (0)

TV Yes (1) No (0)

Fridge Yes (1) No (0)

Bicycle Yes (1) No (0)

Motorbike Yes (1) No (0)

Car Yes (1) No (0)

Boat Yes (1) No (0)

Cart/plough Yes (1) No (0)

**Section H: Intangible costs associated with cholera**

*Note 1: It could be that some patients are still sick from cholera and we may have to follow in order to track costs*.

**Interviewer please start this section of the questionnaire by writing down** the three different starting bids (<1,200, 1,200-5,000, >5,000) based on the annual report on earnings to represent low, middle and high average monthly income in Zambia on separate papers. If mixed then and let the interviewer select one of them randomly. The amount specified on the paper will be the starting bid for this interviwer.

H1. What is the starting bid selected by the interviewer?

Low income data…………………………………..Z MK (1)

Medium income data………………………………….. ZMK (2)

High income data………………………………….. ZMK (3)

H2. Would you like to pay this amount (interviewer spell out the amount in H1) out of your pocket in

addition to your current treatment expenditures in order to avoid the stigma associated with your

cholera disease?

Yes (1) **🡪 Go to H3** No (2) **🡪 Go to H13**

H3. Would you like to pay the double of the amount (*interviewer multiply the amount in H1 by 2 and spell*

*out*) out of your pocket in addition to your current treatment expenditures in order to avoid the stigma

associated with your cholera disease?

Yes (1) **🡪 Go to H5** No (2) **🡪 Go to H4**

H4. What is the exact amount you would be able to pay out-of you pocket between the simple and the double amount (interviewer spell out) in order to avoid stigma associated with your cholera?

ZMK

H5. Would you like to pay the double of the amount (*interviewer multiply the amount you spelled out in*

*H3 by 2 and spell out*) out of your pocket in addition to your current treatment expenditures in order

to avoid the stigma associated with your cholera disease?

Yes (1) **🡪 Go to H7** No (2) **🡪 Go to H6**

H6. What is the exact amount you would be able to pay out-of you pocket between these adjacent simple

and the double amount (interviewer spell out) in order to avoid stigma associated with your

cholera?

ZMK

H7. Would you like to pay the double of the amount (*interviewer multiply the amount you spelled out in H5 by 2 and spell out*) out of your pocket in addition to your current treatment expenditures in order to avoid the stigma associated with your cholera disease?

Yes (1) **🡪 Go to H9** No (2) **🡪 Go to H8**

H8. What is the exact amount you would be able to pay out-of you pocket between these adjacent simple and the double amount (interviewer spell out) in order to avoid stigma associated with your cholera? ZMK

H9. Would you like to pay the double of the amount (*interviewer multiply the amount you spelled out in H7 by 2 and spell out*) out of your pocket in addition to your current treatment expenditures in order to avoid the stigma associated with your cholera disease?

Yes (1) **🡪 Go to H11** No (2) **🡪 Go to H10**

H10. What is the exact amount you would be able to pay out-of you pocket between these adjacent simple and the double amount (interviewer spell out) in order to avoid stigma associated with your cholera? ZMK

H11. Would you like to pay the double of the amount (*interviewer multiply the amount you spelled out in H9 by 2 and spell out*) out of your pocket in addition to your current treatment expenditures in order to avoid the stigma associated with your cholera disease?

Yes (1) **🡪 Go to H12** No (2) **🡪 Go to H12**

H12. What is the exact amount you would be able to pay out-of you pocket between these adjacent simple and the double amount (interviewer spell out) in order to avoid stigma associated with your cholera? ZMK

H13. Would you like to pay less than twice the amount (*interviewer divide the amount you specified in H1 and spell out*) out of your pocket in addition to your current treatment expenditures in order to avoid the stigma associated with your cholera disease?

Yes (1) **🡪 Go to H14** No (2) **🡪 Go to H15**

H14. What is the exact amount you would be like to pay out-of your pocket between these adjacent simple and the less than twice amount (interviewer spell out) in order to avoid stigma associated with your cholera? ZMK

H15. Would you like to pay less than twice the amount (*interviewer divide the amount you specified spelled out in H13 by 2 and spell out*) out of your pocket in addition to your current treatment expenditures in order to avoid the stigma associated with your cholera disease?

Yes (1) **🡪 Go to H16** No (2) **🡪 Go to H17**

H16. What is the exact amount you would be like to pay out-of your pocket between these adjacent simple and the less than twice amount (interviewer spell out) in order to avoid stigma associated with your cholera? ZMK

H17. Would you like to pay less than twice the amount (*interviewer divide the amount you specified spelled out in H15 by 2 and spell out*) out of your pocket in addition to your current treatment expenditures in order to avoid the stigma associated with your cholera disease?

Yes (1) **🡪 Go to H18** No (2) **🡪 Go to H19**

H18. What is the exact amount you would be like to pay out-of your pocket between these adjacent simple and the less than twice amount (interviewer spell out) in order to avoid stigma associated with your cholera? ZMK

H19. Would you like to pay less than twice the amount (*interviewer divide the amount you specified spelled out in H17 by 2 and spell out*) out of your pocket in addition to your current treatment expenditures in order to avoid the stigma associated with your cholera disease?

Yes (1) **🡪 Go to H20** No (2) **🡪 Go to H21**

H20. What is the exact amount you would be like to pay out-of your pocket between these adjacent simple and the less than twice amount (interviewer spell out) in order to avoid stigma associated with your cholera? ZMK

H21. Would you like to pay less than twice the amount (*interviewer divide the amount you specified spelled out in H17 by 2 and spell out*) out of your pocket in addition to your current treatment expenditures in order to avoid the stigma associated with your cholera disease?

Yes **🡪 Go to H22** No (2) **🡪 Go to H22**

H22. What is the exact amount you would be like to pay out-of your pocket between these adjacent simple and the less than twice amount (interviewer spell out) in order to avoid stigma associated with your cholera? ZMK

H23. What is the total household income per month?

0-500 ZMK (1) 501- 1,000 ZMK (2) 1,001-2,000 ZMK (3)

2,001-3,000 ZMK (4) 3,001-4,000 ZMK(5) 4,001-5,000 ZMK (6)

5,001- 7,000ZMK (7) 7,001-10,000 ZMK(8) 10,001-20,000 ZMK (9)

>20,000ZMK (10)

H24. How many people share that income? don’t know=97 ; refusal=99

H25. How many children do you have? don’t know=97 ; refusal=99

**Section I: Costs associated with death from cholera**

Interviewer only proceed to this section, if the patient is dead.

Now, my coming question may evoked a bad memory to you as I will talk about the death of the member of your family who suffered from cholera

I1. What your (the patient) health status today?

Recovered from cholera (1)**🡪Go to** Still sick with cholera (2) **🡪Go to**

Sick with other disease (3) **🡪Go to** Deceased (4)

Other (5) Specify…………………………………………… **🡪Go to**

We are sorry getting to know that the patient passed away because of cholera. We

would like to ask a few more question on his burial. We understand these may evoke in you some negative feelings. Please accept our apologizes. Can we continue?

I2. How many mourners were gathered for the patient burial?

Number (97 = Don’t know)

I3. In total, how much did your family had to pay the mourners?

ZMK (*don’t know=979797; no expense=000000; NA=989898)*

I4. In total, how much did your family had to spend to feed the mourners?

ZMK (*don’t know=979797; no expense=000000; NA=989898)*

I5. In total, how much did your family had to spend on mourners transportation?

ZMK (*don’t know=979797; no expense=000000; NA=989898)*

I6. In total, how much did your family had to spend for the mourners’ lodging?

ZMK (*don’t know=979797; no expense=000000; NA=989898)*

I7. In total, how much did your family had to spend at the morgue?

ZMK (*don’t know=979797; no expense=000000; NA=989898)*

I8. In total, how much did your family had to spend for the transportation of the dead person to the cimetery?

ZMK (*don’t know=979797; no expense=000000; NA=989898)*

I7. In total, how much did your family had to spend for the coffin and the grave?

ZMK (*don’t know=979797; no expense=000000; NA=989898)*

|  |
| --- |

HH1. Time at which interview ended: (HH/MN/SS)

Remarks:…………………………………………………………………………………………………

……………………………………………………………………………………………………………

……………………………………………………………………………………………………………

Date of questionnaire review by the supervisor (if any) (DD/MM/YY)

A6. Supervisor signing…….………………………………………………………………….

Thank you for participating in this research.
